# Inflammation and cardiovascular status impact midazolam pharmacokinetics in critically ill children: An observational, prospective, controlled study

**DOI:** 10.1002/prp2.1004

**Published:** 2022-08-29

**Authors:** Bikalpa Neupane, Hitesh Pandya, Tej Pandya, Rupert Austin, Neil Spooner, James Rudge, Hussain Mulla

**Affiliations:** ^1^ Department of Respiratory Sciences, College of Life Sciences University of Leicester Leicester UK; ^2^ Jenny Lind Children's Hospital Norfolk and Norwich University Hospital NHS Trust Norwich UK; ^3^ Royal Bolton NHS Foundation Trust Farnworth UK; ^4^ BAST Inc Limited Kington UK; ^5^ Spooner Bioanalytical Solutions Limited Hertford UK; ^6^ Neoteryx Torrance California USA; ^7^ Department of Pharmacy University Hospitals of Leicester NHS Trust Leicester UK

**Keywords:** cardiovascular, CRP, inflammation, midazolam, pharmacokinetics, population modeling

## Abstract

Altered physiology caused by critical illness may change midazolam pharmacokinetics and thereby result in adverse reactions and outcomes in this vulnerable patient population. This study set out to determine which critical illness‐related factors impact midazolam pharmacokinetics in children using population modeling. This was an observational, prospective, controlled study of children receiving IV midazolam as part of routine care. Children recruited into the study were either critically‐ill receiving continuous infusions of midazolam or otherwise well, admitted for elective day‐case surgery (control) who received a single IV bolus dose of midazolam. The primary outcome was to determine the population pharmacokinetics and identify covariates that influence midazolam disposition during critical illness. Thirty‐five patients were recruited into the critically ill arm of the study, and 54 children into the control arm. Blood samples for assessing midazolam and 1‐OH‐midazolam concentrations were collected opportunistically (critically ill arm) and in pre‐set time windows (control arm). Pharmacokinetic modeling demonstrated a significant change in midazolam clearance with acute inflammation (measured using C‐Reactive Protein), cardio‐vascular status, and weight. Simulations predict that elevated C‐Reactive Protein and compromised cardiovascular function in critically ill children result in midazolam concentrations up to 10‐fold higher than in healthy children. The extremely high concentrations of midazolam observed in some critically‐ill children indicate that the current therapeutic dosing regimen for midazolam can lead to over‐dosing. Clinicians should be aware of this risk and intensify monitoring for oversedation in such patients.

## Graphical abstract

Pharmacokinetic modelling revealed significantly reduced midazolam clearance in critically ill children with acute inflammation and cardio‐vascular compromise, resulting in up to 10‐fold higher plasma concentrations compared to healthy children.

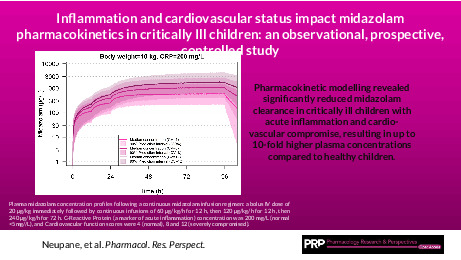

**Abbreviations**
Cl_mid_
: midazolam clearance
CRP: C‐reactive protein
CYP3A4: cytochrome P450 3A4
GABA: gamma‐aminobutyric acid
GOF: goodness of fit
IL‐6: interleukin‐6
PICU: pediatric intensive care unit
PK: pharmacokinetic
UGTs: UDP‐glucuronosyltransferases
VPC: Visual Predictive Check

## 1 INTRODUCTION

Midazolam is a benzodiazepine with sedative, amnesic, and anti‐epileptic properties. An intravenous bolus dose of the drug alleviates symptoms in a matter of minutes and, when administered prior to a short‐lived unpleasant procedure, prevents significant patient distress.^1^

Continuous IV infusion midazolam is commonly used in pediatric critical care to provide sustained patient sedation.^2^ While therapeutically very useful, prolonged midazolam administration often results in drug tolerance and severe adverse reactions including respiratory depression and long‐lived neuro‐psychiatric disturbances on drug withdrawal.^3^, ^4^, ^5^, ^6^, ^7^, ^8^ The frequency of adverse reactions reports suggests that midazolam dosing is not optimal for this population.^9^, ^10^, ^11^ Presently, IV midazolam doses recommended for children are based on weight‐based scaling of doses used in adults.^12^ Conceivably, therefore, personalizing pediatric IV midazolam dosing recommendations, e.g., according to patient genetics or physiology, could optimize its effectiveness and lead to improved long‐term outcomes.

Midazolam acts by potentiating the effects of gamma‐aminobutyric acid (GABA) on GABA_A_ receptors.^13^ It is metabolized in the liver by cytochrome P450 3A4 (CYP3A4) enzyme to an active metabolite (1‐hydroxy midazolam). Glucuronidation of 1‐hydroxy midazolam by UDP‐glucuronosyltransferases (UGTs) generates inactive metabolites that are excreted in the urine. Polymorphisms in CYP3A4 and UGT genes may account for differences in pharmacokinetics in healthy and critically‐ill individuals.^14^ Physiological changes caused by critical illness and treatment interventions could further alter the midazolam exposure‐response relationship.^15^ These include changes in GABA_A_ signal transduction, diminished hepatic blood flow, and altered liver enzyme metabolizing capacity. The latter two factors are potential modifiers of midazolam clearance in the critically ill.^16^, ^17^

This prospective, observational, pharmacokinetic (PK) study explored midazolam disposition in otherwise healthy children undergoing elective surgery (control group) and critically ill patients requiring mechanical ventilation in intensive care. Contemporaneous recruitment of a control group should enable critical illness‐related covariates influencing PK parameters to be identified with greater certainty.

## 2 MATERIALS AND METHODS

### 2.1 Study design and study population

This was a single‐center, observational, prospective study of IV midazolam pharmacokinetics in two groups of children; clinically well children (control group) receiving IV midazolam prior to elective day case surgery and ill, intubated, and ventilated children receiving IV midazolam in intensive care (critically ill group). Children were aged between 1 month (corrected gestational age) and less than 16 years and admitted either for planned surgical procedures requiring general anesthesia or to the pediatric intensive care unit (PICU). Although there was no sample size calculation, a minimum of 50 children were to be recruited with at least 150 PK samples to enable a robust population PK model to be developed.

The study was conducted between January 2015 and September 2016 and complied with the principles of the 1964 Declaration of Helsinki and its later amendments. The study was approved by the East Midlands‐ Derby Research Ethics Committee in England (14/EM/1261) and registered on the EUDRACT database (2014‐004958‐34).

In the control group, midazolam was administered as a single IV ‘bolus’ dose (25‐50 mcg kg^−1^) as part of general anesthetic induction. In the critically ill group, infants and children received an initial 25–50 mcg kg^−1^ bolus dose and were then initiated on a continuous infusion of 50–200 mcg kg^−1^ h^−1^. The target sedation level was assessed and reviewed at least twice daily. The continuous infusion rate was altered according to the unit's algorithm for dose adjustment and titrated to the desired sedation score. Similarly, additional bolus doses were occasionally administered as necessary to achieve and maintain the desired sedation level.

### 2.2 Assessments and endpoints

In the control group, initial PK samples (1 and 2) were taken at various time points, starting from the pre‐dose sample before surgery, during surgery, and after surgery was complete, and surgical drapes covering the child were removed. Subsequent samples (3, 4, and 5) were taken either in the recovery suite or on the wards. PK blood samples were thus obtained for up to 6 h post‐dose.

In the critically ill group, blood samples for PK were either scavenged (from laboratory samples obtained for monitoring patients) or opportunistic (at times when blood samples were being taken for clinical reasons). PK blood samples were obtained for the duration of the period the child was on midazolam infusion and up to 96 h after treatment had stopped.

CRP, liver, and kidney function assessments were determined at least once daily. To measure cardio‐vascular status, a scoring system was developed for this study utilizing data available in the PICU cohort (Table 1). Each variable was scored between 1 and 3 and added together to generate a final score (‘CV score’). Increasing CV score implies worsening cardiovascular function.

**TABLE 1:** Scoring tool for cardiovascular status (CV Score).

Variable	Score		
	1	2	3
Inotrope support	None	1 Inotrope	>1 Inotropes
Base excess	<2	2 to −10	>−10
Volume support (ml/kg)	None	up to 10	>10
Urine output (ml/kg/hr)	>2	1–2	<1

A secondary objective of the study was to validate the volumetric absorptive microsampling system of PK sampling as an alternative to the collection of conventional blood samples in tubes. This volumetric system involved using Mitra® microsampling devices based on VAMS® technology to collect blood for analysis as a dried sample. In both groups of patients, each PK sample was collected as a duplicate: a whole blood wet sample (for centrifugation and processing) and a 10 μl dry sample using the Mitra device with a VAMS system. The validation data for the VAMS system met internationally accepted guideline criteria.^18^, ^19^ A strong correlation was observed in measured concentrations between wet and dry test samples, indicating that VAMS is a suitable technique for use in pediatric clinical studies. The analytical method and results of the validation are presented elsewhere.^20^

A population pharmacokinetic model of midazolam and 1‐hydroxy‐midazolam plasma concentrations was developed using the nonlinear mixed effects modeling program NONMEM (ICON Development Solutions, version 7.4^21^). Full detail on the modeling method is provided in an Appendix S1.

## 3 RESULTS

### 3.1 Patients and data

Thirty‐five patients contributed PK observations from the control group and 54 patients contributed from the critically ill group. Baseline covariates for both groups of patients are listed in Table 2.

**TABLE 2:** Key baseline covariates for all patients.

Demographic/clinical characteristics	Unit	ICU (*n* = 54)		Surgical (*n* = 35)	
		median (range)	mean (IQR)	median (range)	mean (IQR)
Age	Years	1.075 (0.08–16)	3.05 (0.31–3.41)	5.36 (0.62–15.71)	6.22 (3.77–7.98)
Body weight	kg	9.1 (3.0–75.5)	13.7 (4.9–15.6)	19.7 (5.8–59.5)	23.7 (14.8–24.9)
Plasma albumin	g/L	30.0 (13–47)	31.3 (26–36)	Not measured. Set to 32 g/L	
Plasma bilirubin	μmol/L	6.5 (2.0–69.0)	9.72 (3.25–12.75)	Not measured. Set to 5 μmol/L	
Serum creatinine	μmol/L	26.7 (15.5–282)	35.8 (21.9–33.5)	Not measured. Set to 40 μmol/L	
CRP	mg/L	37.5 (5–306)	75.71 (18.5–81.5)	Not measured. Set to 3 mg/L	
CV score	—	7 (4–16)	7.33 (6–9)	Not measured. Set to 4	
Sex		53.7% M	46.2% F	71.4% M	28.6% F

The median (range) age was 22 months (1 month to 15 years). In the control group, 80% of children were over 2 years of age, whereas in the critically ill group the age was skewed toward the younger age group with 67% less than 2 years of age. The body weight ranged from 2.9 to 78.4 kg (median, 13.70 kg) and the BMI ranged from 9.8 to 21.8 kg m^−2^ (median 15.8 kg m^−2^) (Table 2). 81 children were Caucasian, 14 were Asian, 2 were Afro Caribbean and 3 were of mixed ethnicity.

Control group children were mainly admitted for ENT [19 (53%)] and urological [13 (36%)] day case procedures. In the critically ill group, cardiac [23(42%)], respiratory [19 (34%)] and neurological [7 (13%)] disorders were the commonest primary systems causing critical illness.

The mean (range) midazolam bolus dose in the control group was 34.6 (34.6–41.7) mcg kg^−1^. The mean (range) duration and rate of midazolam infusion in the critically ill group was 11.8 h (0.25–372) hours and 129 (8.33–760) mcg kg^−1^ h^−1^, respectively.

**FIGURE 1:**
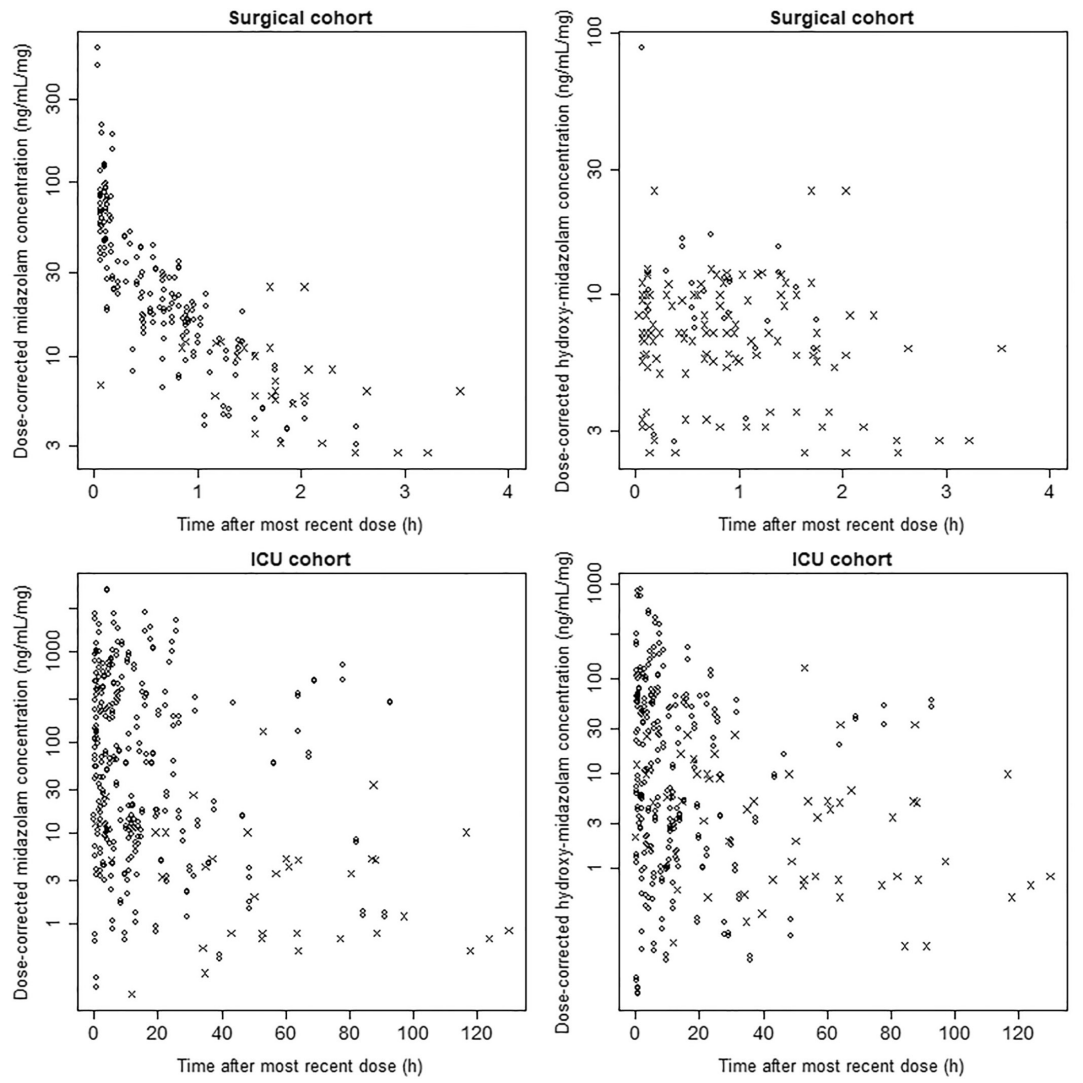
Observed dose‐corrected midazolam and hydroxy‐midazolam concentrations (observed concentration/most recent midazolam dose size) in plasma versus time after most recent dose. Open circles are from quantified observations. Crosses are from observations <LLOQ and are depicted at LLOQ/most recent dose size.

In total, 626 plasma midazolam and 628 1‐hydroxy midazolam PK samples were included in the modeling dataset, of which 119 (19%) and 318 (51%) were below LLOQ, respectively. Mean (range) midazolam concentrations in the control group were 28 (5–356) ng/ml and in the critically ill group was 332 (5–1987) ng ml^−1^. Mean (range) 1‐OH Midazolam concentrations in the control group were 9 (5–64) ng ml^−1^ and in the critically ill group was 56 (5–1507) ng ml^−1^. Scatter plots of dose‐corrected midazolam and 1‐hydroxy midazolam concentrations are presented in Figure 1. The simple pattern of decaying midazolam concentration seen in the control group is not reflected in the critically ill group due to the much wider range of dosing regimens (often a mixture of bolus and infusion doses) used in intensive care patients.

Mean (range) CRP in the critically ill group was 375.5 (3–306) mg L^−1^. This suggests a significant proportion of patients were septic. CRP was assumed to be normal (≤3 mg L^−1^) in the control group. The mean (range) CV score was 7 (4–12) in the critically ill group. Control group patients did not have cardiovascular dysfunction or any requirement for inotropic support and therefore a fixed score of 4 was imputed during model development.

### 3.2 PK model development

A two‐compartmental structural model for midazolam, with a single additional compartment for 1‐hydroxy midazolam, described the observed PK data satisfactorily. Due to structural non‐identifiability of the volume of distribution of the 1‐hydroxymidazolam compartment, it was set equal to that of the midazolam central compartment. Interindividual variability (IIV) could only be optimally quantified for midazolam clearance (CLmid), 1‐hydroxy midazolam clearance (CLHmid), and volume of distribution of the central compartment (*V*_c_). Separate residual error models were applied for the two cohorts and the use of the third variance of residual error was utilized for 1‐hydroxy‐midazolam observations across both cohorts. Due to the wide range of body weights across the two cohorts, body weight‐based allometry was tested upfront during the development of the base model, and was found to be highly significant on CLmid (with a fixed exponent of 0.75) but not on *V*_c_. Testing of the incorporation of a published maturation function for midazolam clearance (a Hill‐type equation that describes the influence of age on midazolam clearance^22^) produced a reduction in OFV of 6.40 units, which did not reach the required level of statistical significance. The goodness of fit plots (GOF) and Visual Predictive Check (VPC) from the base model were satisfactory; GOF plots (Appendix S2, Figures S1–S6) revealed no indication of substantial bias in model residuals and VPC (Appendix S2, Figures S8 and S9) showed good concordance between observations and simulations from the model for both midazolam and hydroxy‐midazolam.

Following the selection of the base PK model, exploratory plots of the IIV versus patient covariates revealed four visually compelling relationships, all involving CLmid: CRP, CV score, serum albumin, and total bilirubin in the blood (Appendix S2, Figure S‐7). CRP entered the model as the most significant covariate in the first step (ΔOFV = −49.2) and CV score entered the model as the most significant covariate in the second step (ΔOFV = −12.8). Neither serum albumin nor total bilirubin ws significant in the third step. Hence, the final PK model contains the influence of both CRP and CV scores on CLmid, where an increase in each of the covariates leads to a reduction in midazolam clearance. The structure of the final PK model is shown in Appendix S2, Figure S‐18, and the associated parameter estimates are listed in Table 3. GOF plots (Appendix S2, Figures S10 to S15) and VPC (Appendix S2, Figures S16 and S17) from the final model were satisfactory. A full description of the model results can be found in Appendix S2.

**TABLE 3:** Parameter estimates for final PK model.

Parameter	Unit	Estimate	RSE [%]^a^	LLCI^b^	ULCI^c^	Description
Fixed effects (THETA)						
CL_mid_	L/h	51.2	11.6	39.5	62.8	Midazolam clearance for the subject with 70 kg body weight, CRP of 3 mg/L, and HD score of 4
*V*_c_	L	9.82	28.9	4.26	15.4	Volume of distribution of midazolam and hydroxy‐midazolam central compartments
*Q*	L/h	13.7	8.62	11.4	16.0	Inter‐compartmental clearance of midazolam between central and peripheral compartments
*V*_p_	V	9.89	9.84	7.98	11.8	Volume of distribution of midazolam peripheral compartment
CL_Hmid_	L/h	29.4	31.8	11.0	47.7	Hydroxy‐midazolam clearance
KMET	h^−1^	0.448	18.0	0.290	0.606	Rate constant for conversion of midazolam to hydroxy‐midazolam
*θ*_CRP_	L/mg	−0.00569	16.4	−0.00751	−0.00386	Influence of CRP on CL_mid_
*θ*_HD_	HD units^−1^	−0.147	18.9	−0.201	−0.0922	Influence of HD score on CL_mid_
Random effects: Inter‐individual variability (OMEGA)						
CL_mid_ (*ω* ^2^) CV^d^ Shrinkage	— % %	0.320 61.4 8.2	17.3	0.212 48.5	0.429 73.1	Variance of exponential IIV on CL_mid_
*V*_c_ (*ω* ^2^) CV^d^ Shrinkage	— % %	2.12 270 17.1	29.5	0.894 120	3.34 521	Variance of exponential IIV on *V*_c_
CL_Hmid_ (*ω* ^2^) CV^d^ Shrinkage	— % %	2.81 395 14.7	25.5	1.40 175	4.22 819	Variance of exponential IIV on CL_Hmid_
*V*_c_/CL_Hmid_ covariance	—	2.17	29.4	0.918	3.42	Covariance between IIV on *V*_c_ and IIV on CL_Hmid_
Residual error^e^ (SIGMA)						
*σ* ^2^ (mid,surg) CV	— %	0.0444 21.3	15.3	0.0311 17.8	0.0576 24.4	Variance of additive residual error for log‐transformed midazolam concentration in Group 1
*σ* ^2^ (mid,ICU) CV	— %	0.342 63.9	7.28	0.293 58.4	0.391 69.1	Variance of additive residual error for log‐transformed midazolam concentration in Group 2
*σ* ^2^ (Hmid) CV	— %	0.299 59.0	6.31	0.262 54.7	0.336 63.2	Variance of additive residual error for log‐transformed hydroxy‐midazolam concentration

^a^ RSE = relative standard error (100·SE/estimate).

^b^ LLCI = lower limit of 95% confidence interval (estimate ‐ 1.96·SE).

^c^ ULCI = upper limit of 95% confidence interval (estimate +1.96·SE).

^d^ Coefficient of variation (CV) calculated as 100·SQRT(EXP(*ω* ^2^) − 1). The confidence intervals of CV are derived through the transformation of confidence intervals of *ω* ^2^.

^e^ Both the observations and the model predictions were log‐transformed and an additive residual error model was used. This is equivalent to an exponential residual error model on untransformed data, and the coefficient of variation (CV) was calculated as 100·SQRT(EXP[*σ* ^2^] − 1). The confidence intervals of CV are derived through the transformation of confidence intervals of *σ* ^2^.

### 3.3 Simulations

**FIGURE 2:**
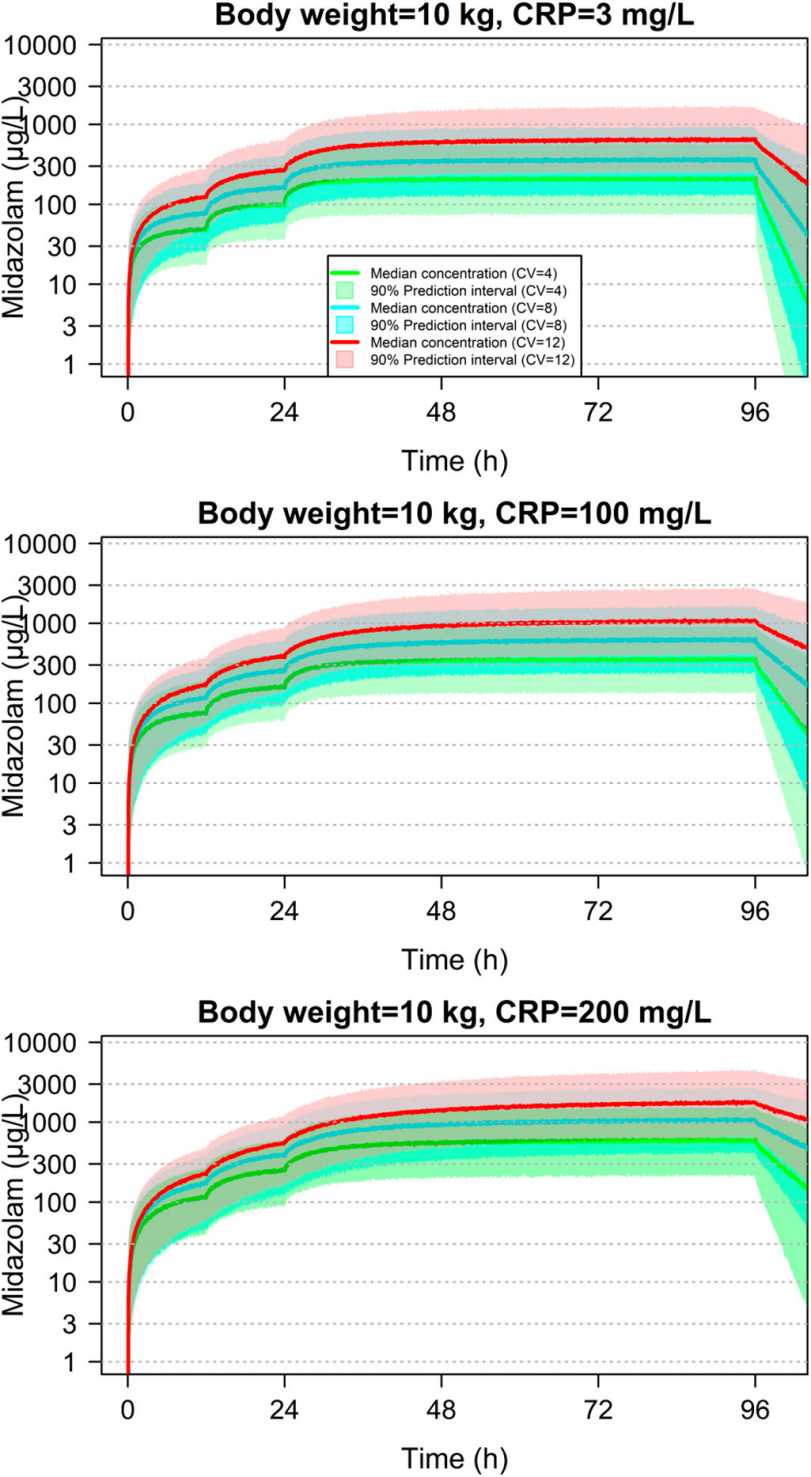
Graphical displays of simulation outputs following a continuous midazolam infusion regimen in a child of bodyweight 10 kg: a bolus IV dose of 20 μg/kg immediately followed by continuous infusions of 60 μg/kg/h for 12 h, then 120 μg/kg/h for 12 h, then 240 μg/kg/h for 72 h. CRP concentrations were 3, 100, and 200 mg/L, and CV scores were 4, 8, and 12. CRP varies within the 3 plots in each panel and CV score varies within each plot. (Additional simulations can be found in Appendix S2).

The final model incorporating bodyweight, CRP and CV score effects CLmid was used to simulate PK profiles of midazolam following a typical continuous infusion dosing regimen (Figure 2; Appendix S2, Figure S‐18). A tabulated version of the data with median and 95% prediction intervals can be found in the Appendix S2.

The simulations predict a significant increase in steady state midazolam concentrations with rising CRP and CV score. Compared to baseline CRP (<3 mg L^−1^), a rise >200 mg L^−1^ results in a 3‐fold increase in steady state concentrations. A similar fold increase is seen when the CV score increases from baseline (=4) to 12. A combination of high CRP > 200 mg L^−1^ and significant cardiovascular compromise (CV score = 12) results in a massive 7–10 fold increase in steady state concentrations.

## 4 DISCUSSION

It is well established that altered physiology caused by critical illness alters the PK and pharmacodynamics of many drugs as a result of disrupted one or more ‘ADME’ processes.^23^, ^24^ In this prospective population PK study, we show that markers of systemic inflammation (CRP) and cardiovascular function (CV score) are associated with reduced midazolam clearance in critically ill children. Similar findings have been reported previously but unlike previous studies, the inclusion of healthy children in our study increases the certainty of the findings.^16^, ^17^

C‐reactive protein (CRP) is an acute phase reactant that increases during periods of inflammation, e.g., due to sepsis or trauma. Its concentration in blood correlates with concentrations of interleukin‐6 (IL‐6) a pro‐inflammatory cytokine that is known to induce CRP. The PK model developed herein estimates that critically ill children with a CRP > 100 ng ml^−1^ have almost a 50% reduction in midazolam clearance (Cl_mid_) and those with a CRP > 200 ng ml^−1^ Cl_mid_ reduces around 70% compared to otherwise healthy children with a CRP <3 ng ml^−1^.

Reduced urine output, treatment to support blood pressure (inotropic drugs such as dopamine and boluses of fluid), and increased acid (negative base excess) production are all features of depressed cardiovascular function. In this study, these variables were used to develop a score to provide an integrated measure (CV score, Table 1) of cardiovascular dysfunction. Children with a CV score of 12 are estimated to have an almost 75% reduction in midazolam clearance compared to those with a normal (=4) CV score. Model simulations predict that a combination of a CRP > 200 ng ml^−1^ and CV score = 12 will result in median midazolam concentrations between 1000 and 3000 ng ml^−1^ (higher in children than infants) when administered continuous IV midazolam infusion at recommended dosing rates. Although no pharmacodynamics assessments were included in our analysis and therefore the clinical impact of increasing midazolam exposure cannot be quantified, previous studies have suggested that the therapeutic range is in the region of 200–800 ng ml^−1^, but concentrations as high as 2000 ng ml^−1^ were recorded in several of the critically ill children in this study.^3^, ^25^, ^26^

Vet et al (2012) investigated the effect of inflammation and disease severity on midazolam pharmacokinetics in 21 critically ill children.^27^ No correlation was found between CRP and clearance, although clearance was significantly lower in children with multi‐organ failure (assessed using the Pediatric Logistic Organ Dysfunction [PELOD] score). In a subsequent larger cohort study of 83 critically ill children aged 1 day to 7 years, a CRP of 300 mg L^−1^ was associated with a 64.5% lower clearance than a CRP of 10 mg L^−1^ and three failing organs were associated with a 35% lower clearance compared with one failing organ.^17^ The investigators interpreted the effect of failing organs as largely the result of altered hepatic blood flow.

In healthy individuals, midazolam has a low/intermediate hepatic extraction ratio. Consequently, midazolam clearance in health is largely dependent on liver CYP3A4 activity and less influenced by changes in liver blood flow.^28^ Hence, given the observed negative correlation between CRP and midazolam clearance, reduced CYP3A4 enzyme activity secondary to inflammation could account for reduced midazolam clearance in pediatric critical illness. More specifically, interleukin‐6 (IL‐6) is known to promote CRP synthesis and, in contrast, strongly inhibits hepatic CYP3A4 activity.^29^, ^30^, ^31^, ^32^, ^33^ Whether CYP3A4 inhibition wholly accounts for reduced midazolam clearance in pediatric critical illness is unclear. The negative correlation between CV score and midazolam clearance (a threefold increase in midazolam concentrations associated with an increase in CV score from 4 to 12 is consistent) suggests that hepatic vein blood flow is also a critical factor. Ischemia‐induced hepatocyte injury could directly reduce CYP3A4 capacity or as a consequence of IL‐6 release indirectly reduce CYP3A4 activity. Reduced substrate delivery to hepatocytes due to reduced hepatic blood flow is a more likely explanation, as no patient in this study had clinical evidence of severe liver damage.

The clinical implication here is that intensivists need to be cognizant of the impact of rising CRP and poor cardiovascular status on the PK of midazolam and other CYP3A4 substrates too. Inflammation and cardiovascular status are dynamic in a critically ill patient and therefore may account for the large intra‐individual variability. Such pathophysiological changes in critically ill patients can modify exposure to a previously stable drug regimen, possibly resulting in either an increased incidence of adverse reactions or a lack of efficacy. Extrapolating these results to other CYP3A4 substrates needs to be confirmed through clinical studies, but the impact of inflammation, for example, needs to be considered when unexpected variations in the blood concentrations of a CYP3A4 substrate occur in the absence of direct drug–drug interactions.

Despite clearly associating two pathophysiological variables with midazolam clearance in critically ill children, in this study, nearly 60% of the variance in clearance was assigned to inter‐individual variability, i.e. remains unexplained. This suggests a need for further studies to investigate and model the influence of genetic and other factors on midazolam. However, it also suggests that clinical outcomes may be significantly improved through a more personalized approach to midazolam dosing in pediatric intensive care units.

Some limitations of our study should be acknowledged. Only a single bolus dose was administered to the healthy cohort whereas the critically ill child received prolonged, continuous infusions as well as multiple bolus doses. Thus, the pooled data from the two populations were unbalanced with respect to the dosing profile. However, single‐dose midazolam PK is predictive of steady state concentrations following continuous infusion and therefore the pooled analysis is unlikely to be biased. Although the inclusion of healthy children in the study adds weight to the identification of influential covariates, it does not in itself definitively establish a causal relationship between inflammation (rise in CRP) and CYP3A4 suppression. Future proof of mechanism study could perhaps utilize IL‐6 blocking antibodies to provide definitive proof. Finally, our cardiovascular status scoring tool was a bespoke development for this study and not a validated tool. Nevertheless, the inclusion of inotropes, fluid resuscitation, base excess, and urine output in the scoring calculation are considered standard clinical indicators and taken together, sensitive to changing cardiovascular status.

## 5 CONCLUSIONS

In this population PK study of midazolam, we have shown that acute systemic inflammation and cardiovascular status significantly influence midazolam clearance in children. This finding has implications for other drugs metabolized by CYP3A4 too. This confirms the findings of previous studies, but the inclusion of a control group of healthy children gives greater confidence in the identification of these two influential pathophysiological effects. Even with guideline‐recommended dosing regimens, significantly reduced midazolam clearance will result in supra‐therapeutic systemic concentrations and substantially increase the risk of adverse reactions that can impact short and long‐term clinical outcomes. Intensive care clinicians need to be mindful of this; intensify monitoring of such patients with a frequent assessment of sedation scores, arousal scales, daily interruption of sedatives, and monitoring for delirium to avoid overdosing.

## AUTHOR CONTRIBUTIONS

BN, HP, and HM had full access to all the data in the study and take responsibility for the integrity of the data and the accuracy of the data analysis. Study concept and design: BN, HP, NS, JR, and HM. Analysis and interpretation of data: BN, HP, TP, RA, and HM. Drafting of the manuscript: BN, TP, HP, RA, and HM. Critical revision of the manuscript for important intellectual content: NS and JR.

## CONFLICT OF INTEREST

The authors declare no conflicts of interest.

## ETHICS APPROVAL

The study was approved by the East Midlands‐ Derby Research Ethics Committee in England (14/EM/1261).

## CLINICAL TRIAL REGISTRATION

EUDRACT: 2014‐004958‐34. Date of registration: 30th/Jan/2015

## PATIENT CONSENT STATEMENT

Written informed consent (and assent where appropriate) was obtained from each patient's parent(s)/legal guardian before any procedures or assessments were performed.

## Supporting information


Appendix S1

Appendix S2

## Data Availability

The data that support the findings of this study are available on request from the corresponding authors.
