# Supplementary material for: Inflammation and cardiovascular status impact midazolam pharmacokinetics in critically ill children: An observational, prospective, controlled study
Source: Pharmacol Res Perspect. 2022 Aug 29;10(5):e01004. doi: 10.1002/prp2.1004 (PMC9422629; doi:10.1002/prp2.1004)
Supplement: Supplementary file 1 — Appendix S1 [file PRP2-10-e01004-s002.docx]

**Supplementary File 1**

**Inflammation and cardiovascular status impact midazolam pharmacokinetics in critically Ill children: an observational, prospective, controlled study**

Bikalpa Neupane MBBS, MRCPCH(UK) (1,6), Hitesh Pandya MD MRCP (UK) (1), Tej Pandya MBChB (2), Rupert Austin PhD (3), Neil Spooner PhD (4), James Rudge PhD (5), Hussain Mulla PhD (1,7)

1. Department of Respiratory Sciences, College of Life Sciences, University of Leicester, University Road, Leicester LE1 7RH, UK
2. Royal Bolton NHS Foundation Trust, Farnworth, BL4 0JR, England
3. BAST Inc Limited, 61 Bridge St, Kington, HR5 3DJ, England
4. Spooner Bioanalytical Solutions Limited, Hertford, SG13BQ, England.
5. Neoteryx, Torrance, California, 90501, United States of America.
6. Jenny Lind Children’s Hospital, Norfolk and Norwich University Hospital NHS Trust, Norwich, NR4 7UY, England
7. Department of Pharmacy, University Hospitals of Leicester NHS Trust, Leicester LE15WW, England

Midazolam and 1-hydroxymidazolam concentration-time data were analyzed using nonlinear mixed effects modelling implemented with the Monte-Carlo importance sampling (IMP) estimation method [1] in NONMEM software [2]. All numerical and graphical analyses of model outputs were implemented using R software [3]. Concentration observations below the lower limit of quantification (LLOQ = <10ng/ml) were incorporated into the parameter estimation with use of the M3 likelihood method [4]. Models were fit to the natural logarithm of the observed data using an additive model for the residual error. Since the data from the two bioanalytical assay types (wet sample and VAMS) were highly correlated with no significant bias, the data from both assays were modelled simultaneously.

Model development proceeded in three steps: (1) selection of a structural model and an error model leading to a base model, (2) covariate analysis, (3) internal validation of the model. During development of the base model one- and two-compartment models for midazolam were tested. A single compartment for 1-hydroxy-midazolam was utilised throughout as it has been shown that this provides a sufficient description for this species [5]. Quantification of inter-individual variability (IIV) was tested on clearance and volume parameters and was assumed to follow a log-normal distribution. Body weight-based allometric scaling of clearance and volume parameters was tested according to:

$$P_{i}=\theta_{TV}\cdot\left( \frac{{WGHT}_{i}}{70} \right)^{\theta_{wght}}\cdot exp \left( \eta\right)$$

Where Pi denotes the individual PK parameter value in patient i, θTV is the estimated typical value of P in the population, WGHTi is the body weight in patient i, θwght is the allometric exponent parameter (models were tested where θwght was fixed to values of 0.75 (for clearance) and 1.0 (for volume) [6] and where the optimal value of θwght was estimated), and η denotes a normally distributed random variable with mean of 0 and variance ω2, which is used for quantification of IIV.

The published maturation function for midazolam elimination (10) was tested through incorporation into the equation for midazolam clearance:

$${CLmid}_{i}={CLmid}_{TV}\cdot\left( \frac{{WGHT}_{i}}{70} \right)^{\theta_{wght}}\cdot\left( \frac{{PMA}^{H}}{{PMA}^{H}+{TM50}^{H}} \right)\cdot exp \left( \eta\right)$$

Where *CLmid_i_* is the midazolam clearance in a particular individual, *CLmid* is the estimated typical value of midazolam clearance in the population, *PMA* is the postmenstrual age of the individual (40+age in week since birth), *TM50* is the maturation half-time, and *H* is the Hill coefficient. Models were tested where *PMA* and *H* were fixed to their published values (73.6 weeks and 3.0 (10)) and where these parameters were estimated.

Once the base model was selected, covariates were tested for their influence on those PK parameters with quantified IIV. Exploratory scatter plots of individual parameter estimates from the base model against patient covariates (Continuous: age, body weight, serum albumin, alanine aminotransferase, total blood bilirubin, serum creatinine, C-reactive protein, CV score. Categorical: flag for co-administration of enzyme inducers, flag for co-administration of enzyme inhibitors, sex) were used to pre-select covariates for testing in a formal stepwise forward-addition covariate search. In the covariate search the continuous covariates were tested according to:

$$P_{i}=\theta_{TV}\cdot exp\left( \theta_{cov}\cdot\left( {COV}_{i}-{COV}_{std} \right) \right)\cdot exp \left( \eta\right)$$

Where COVi denotes the value of the covariate in patient i, COVstd is a standard value of the covariate (median or mode, depending on covariate) used for centering the covariate relationship, and θcov is the estimated parameter describing the influence of the covariate on the PK parameter.

When testing models containing additional parameters, a reduction in objective function value (OFV) of greater than 6.64 units (for one additional parameter) was required to declare significance at P<0.01 according to the χ2 distribution and the likelihood ratio test. Decisions on model acceptance were also informed using goodness-of-fit (GOF) plots (stratified by surgical / ICU cohort), and other standard model acceptance criteria including successful NONMEM covariance procedure and ETA shrinkage <30% [7,8]. Internal validation of the base model and final covariate model was accomplished through visual predictive check (VPC) using 1000 repeat simulations of the entire dataset [9].

**PK Simulation**

Numerical integration of the final model was used to simulate PK profiles of midazolam following a body weight-based dosing regimen and with various patient covariates. The dosing regimen involved a bolus IV dose of 20 µg/kg immediately followed by continuous infusions of 60 µg/kg/h for 12 h, then 120 µg/kg/h for 12 h, then 240 µg/kg/h for 72 h. Body weights of the virtual patients were 3.5, 10, 20 and 60 kg. CRP levels were 3, 100 and 200 mg/L, and HD scores were 4, 8 and 12. All combinations of body weight, CRP concentration and CV score were simulated (36 scenarios). Each scenario was simulated using 1000 virtual patients with inter-individual and residual variability included as defined by the random effect parameter estimates of the final model.

**Supplementary References**

1. Gibiansky, Leonid, Ekaterina Gibiansky, and Robert Bauer. Comparison of Nonmem 7.2 estimation methods and parallel processing efficiency on a target-mediated drug disposition model. Journal of pharmacokinetics and pharmacodynamics 39.1 (2012): 17-35.

2. NONMEM version 7.4.3 (ICON Development Solutions, Elliott City, MD, USA).

3. R (Version 3.6.1) Development Core Team (2008). R: A language and environment for statistical computing. R Foundation for Statistical Computing, Vienna, Austria. ISBN 3-900051-07-0, URL http://www.R-project.org.

4. Bergstrand, Martin, and Mats O. Karlsson. Handling data below the limit of quantification in mixed effect models. The AAPS journal 11.2 (2009): 371-380.

5. Ahsman, Maurice J., et al. Population pharmacokinetics of midazolam and its metabolites during venoarterial extracorporeal membrane oxygenation in neonates. Clinical pharmacokinetics 49.6 (2010): 407-419.

6. Anderson, Brian J., and Nicholas HG Holford. Mechanism-based concepts of size and maturity in pharmacokinetics. Annu. Rev. Pharmacol. Toxicol. 48 (2008): 303-332.

7. Byon, W., et al. Establishing best practices and guidance in population modeling: an experience with an internal population pharmacokinetic analysis guidance. CPT: pharmacometrics & systems pharmacology 2.7 (2013): 1-8.

8. Savic, R.M. & Karlsson, M.O. Importance of shrinkage in empirical bayes estimates for

diagnostics: problems and solutions. AAPS J. (2009): 11, 558–569.

9. Bergstrand, Martin, et al. Prediction-corrected visual predictive checks for diagnosing nonlinear mixed-effects models. The AAPS journal 13.2 (2011): 143-151.

10. Anderson BJ and Larsson P (2011), A maturation model for midazolam clearance. Pediatric Anesthesia 21(3):302-308.
