# Supplementary material for: Inflammation and cardiovascular status impact midazolam pharmacokinetics in critically ill children: An observational, prospective, controlled study
Source: Pharmacol Res Perspect. 2022 Aug 29;10(5):e01004. doi: 10.1002/prp2.1004 (PMC9422629; doi:10.1002/prp2.1004)
Supplement: Supplementary file 2 — Appendix S2 [file PRP2-10-e01004-s001.docx]

**Supplementary File 2**

**Inflammation and cardiovascular status impact midazolam pharmacokinetics in critically Ill children: an observational, prospective, controlled study**

Bikalpa Neupane MBBS, MRCPCH(UK) (1,6), Hitesh Pandya MD MRCP (UK) (1), Tej Pandya MBChB (2), Rupert Austin PhD (3), Neil Spooner PhD (4), James Rudge PhD (5), Hussain Mulla PhD (1,7)

1. Department of Respiratory Sciences, College of Life Sciences, University of Leicester, University Road, Leicester LE1 7RH, UK
2. Royal Bolton NHS Foundation Trust, Farnworth, BL4 0JR, England
3. BAST Inc Limited, 61 Bridge St, Kington, HR5 3DJ, England
4. Spooner Bioanalytical Solutions Limited, Hertford, SG13BQ, England.
5. Neoteryx, Torrance, California, 90501, United States of America.
6. Jenny Lind Children’s Hospital, Norfolk and Norwich University Hospital NHS Trust, Norwich, NR4 7UY, England
7. Department of Pharmacy, University Hospitals of Leicester NHS Trust, Leicester LE15WW, England

**Results:**

**PK Model Development**

A two-compartmental structural model for midazolam, with a single additional compartment for 1-hydroxy midazolam, described the observed PK data satisfactorily. Parameters where IIV was found to be optimally quantified and with acceptable ETA shrinkage were midazolam clearance (CLmid), 1-hydroxy midazolam clearance (CLHmid) and volume of distribution of the central compartment (Vc). Significant correlation between individual ETA estimates on CLHmid and Vc was apparent so a covariance term was introduced (117 unit reduction in OFV). Due to the wide range of body weight, it was decided to test body weight-based allometry for influence on CLmid and Vc during development of the base model rather than in the later covariate search. Inclusion of body weight-based allometry on the CLmid parameter using a fixed exponent of 0.75 led to a highly significant 42.6 unit reduction in OFV. Estimation of the exponent led to a further 3.7 unit reduction in OFV which did not reach the criterion for significance, hence the exponent remained fixed to 0.75 in all further models. Body weight based allometry on Vc did not achieve significance. Exploration of the residual error model revealed much larger residual error among the ICU cohort and the use of separate variances of residual error for midazolam observations for the two cohorts was highly significant (124 unit reduction in OFV). A third variance of residual error was utilised for hydroxy-midazolam observations across both cohorts. GOF plots and VPC from the base model were satisfactory; GOF plots (Figures S-I to S-VI) revealed no indication of substantial bias in model residuals and VPC (Figures S-VII to S-VIII) showed good concordance between observations and simulations from the model for both midazolam and hydroxy-midazolam.

Following selection of the base PK model, exploratory plots of the IIV (ETA values associated with CLmid, Vc and CLHmid) versus patient covariates revealed four visually compelling relationships, all involving CLmid: CRP, CV score, serum albumin and total bilirubin in blood (Figure S-IX). These covariates were tested in a stepwise forward-addition procedure where CRP entered the model as the most significant covariate in the first step (ΔOFV=-49.2) and CV score entered the model as the most significant covariate in the second step (ΔOFV=-12.8). Neither serum albumin or total bilirubin were significant in the third step. Hence, the final PK model contains the influence of both CRP and CV score on midazolam clearance, where an increase in each of the covariates leads to a reduction in clearance. GOF plots (Figures S-X to S-XV) and VPC (Figures S-XVI to S-XVII) from the final model were satisfactory. The structure of the final PK model is shown in Figure XVIII, and the associated parameter estimates are listed in Table 3.

It should be noted that with hydroxy-midazolam, given the high proportion of observations below the lower limit of quantification, the GOF plots are of limited diagnostic utility since residuals are not calculable for 51% of the observations. Hence, the calculable residuals could have a tendency for bias because they will tend to have arisen mostly from those subjects with higher than average concentrations. Consequently, more emphasis should be placed on quality of VPC where both quantified observations and observations below lower limit of quantification have been included in the assessment.

# Supplementary Figures

**Abbreviations**

DV Dependent variable (midazolam / hydroxy midazolam concentration)

CWRES Conditional weighted residuals

PRED Population prediction

IPRED Individual prediction

TAD Time after most recent dose


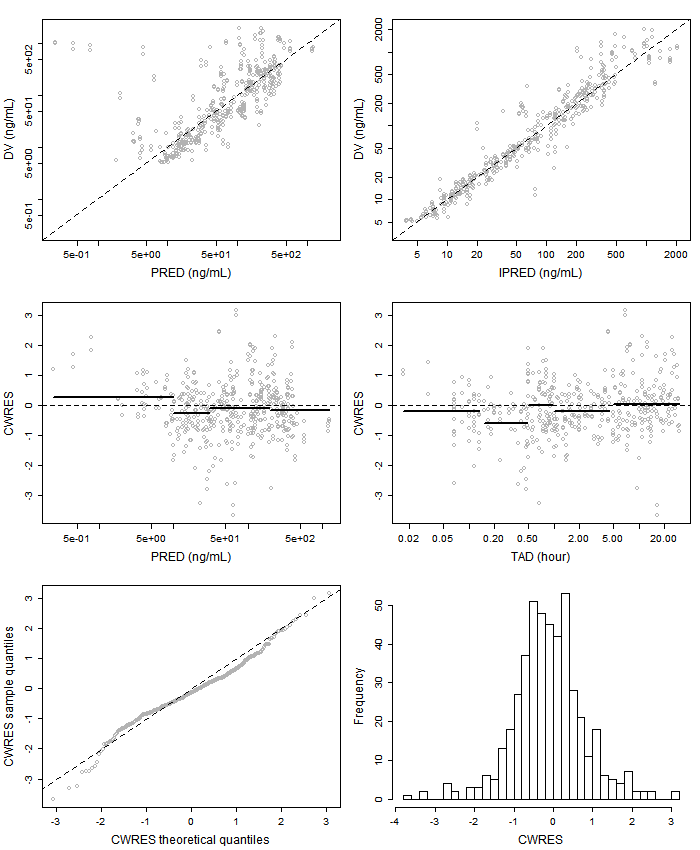


**Figure S-I:** Goodness of fit plots for midazolam observations from base PK model*.* All data included. Bold horizontal lines show the arithmetic mean of CWRES within the bin of independent variable (PRED, IPRED, TAD).


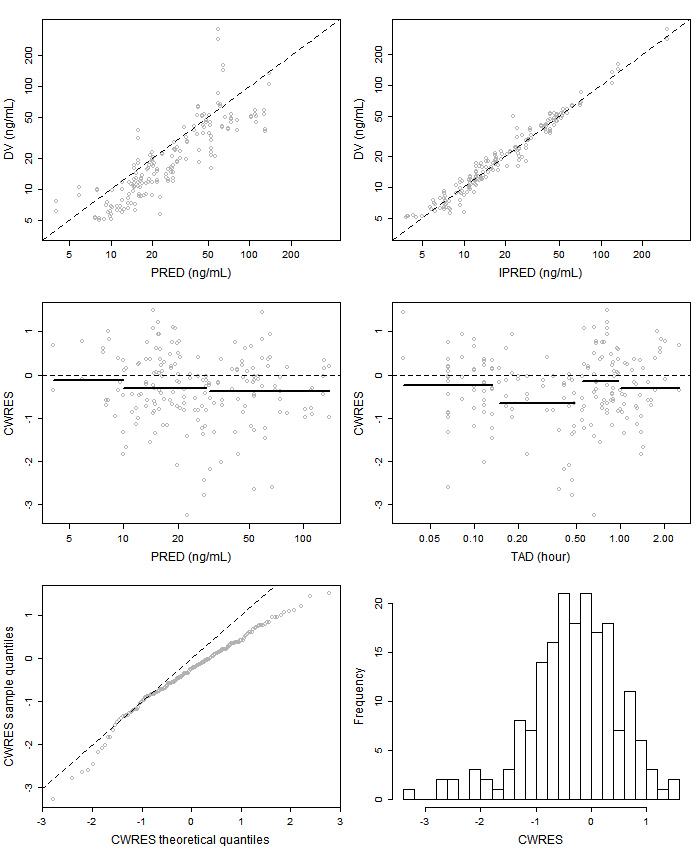


**Figure S-II:** Goodness of fit plots for midazolam observations from base PK model*.* Only data from surgical cohort included. Bold horizontal lines show the arithmetic mean of CWRES within the bin of independent variable (PRED, IPRED, TAD).


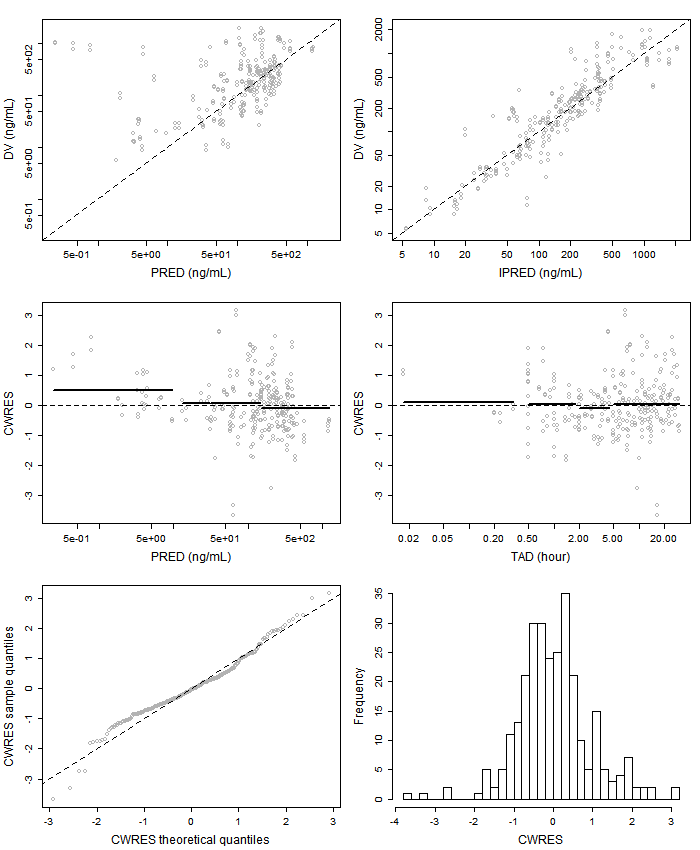


**Figure S-III:** Goodness of fit plots for midazolam observations from base PK model*.* Only data from ICU cohort included. Bold horizontal lines show the arithmetic mean of CWRES within the bin of independent variable (PRED, IPRED, TAD).


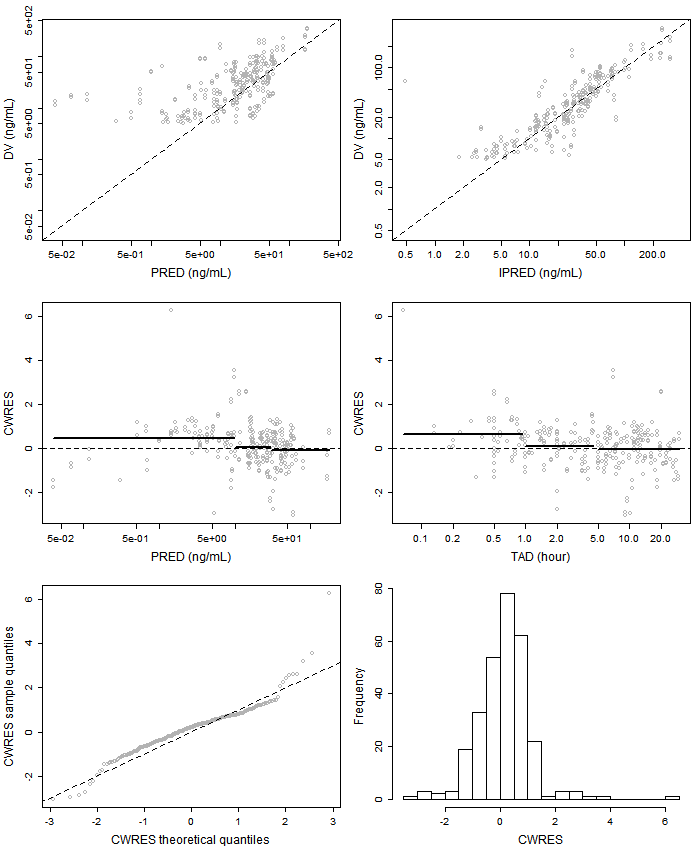


**Figure S-IV:** Goodness of fit plots for hydroxy-midazolam observations from base PK model*.* All data included. Bold horizontal lines show the arithmetic mean of CWRES within the bin of independent variable (PRED, IPRED, TAD).


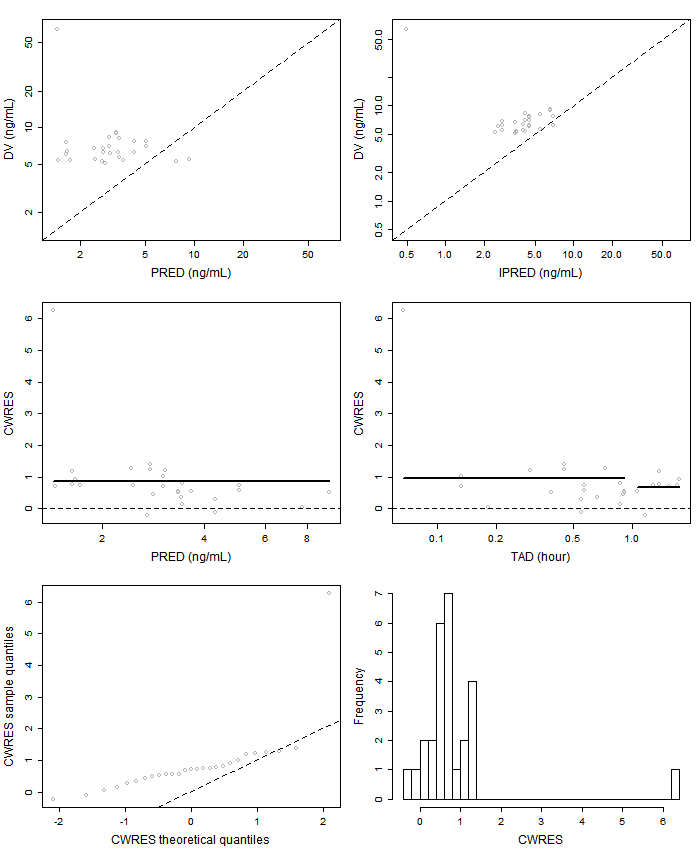


**Figure S-V:** Goodness of fit plots for hydroxy-midazolam observations from base PK model*.* Only data from surgical cohort included. Bold horizontal lines show the arithmetic mean of CWRES within the bin of independent variable (PRED, IPRED, TAD). Very few quantified hydroxy-midazolam observations in surgical cohort.


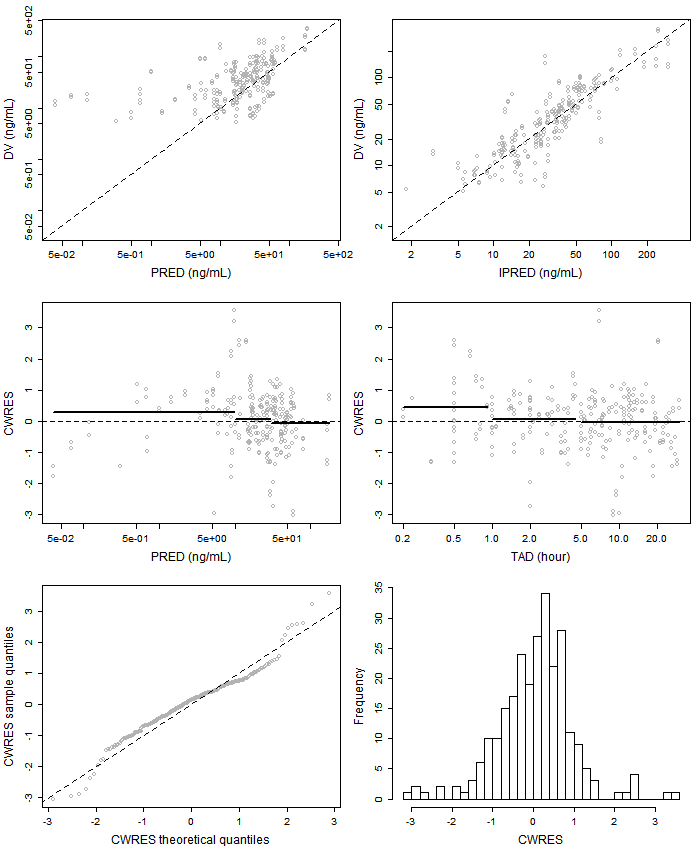


**Figure S-VI:** Goodness of fit plots for hydroxy-midazolam observations from base PK model*.* Only data from ICU cohort included. Bold horizontal lines show the arithmetic mean of CWRES within the bin of independent variable (PRED, IPRED, TAD).


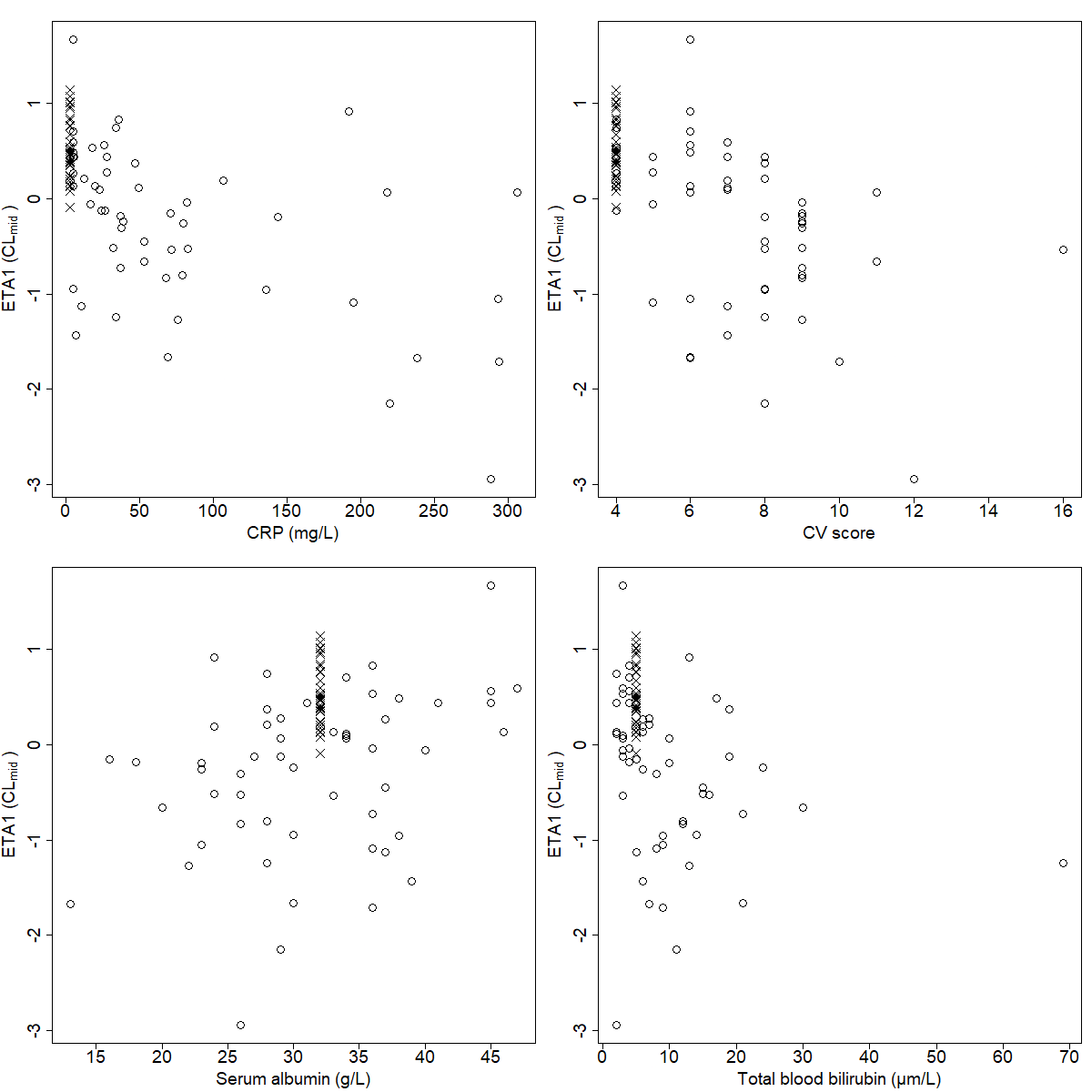


**Figure S-VII:** Inter-individual variability (ETA) in midazolam clearance from base PK model versus CRP, CV score, serum albumin and total blood bilirubin. Open circles are data from the surgical cohort. Crosses are data from the ICU cohort.


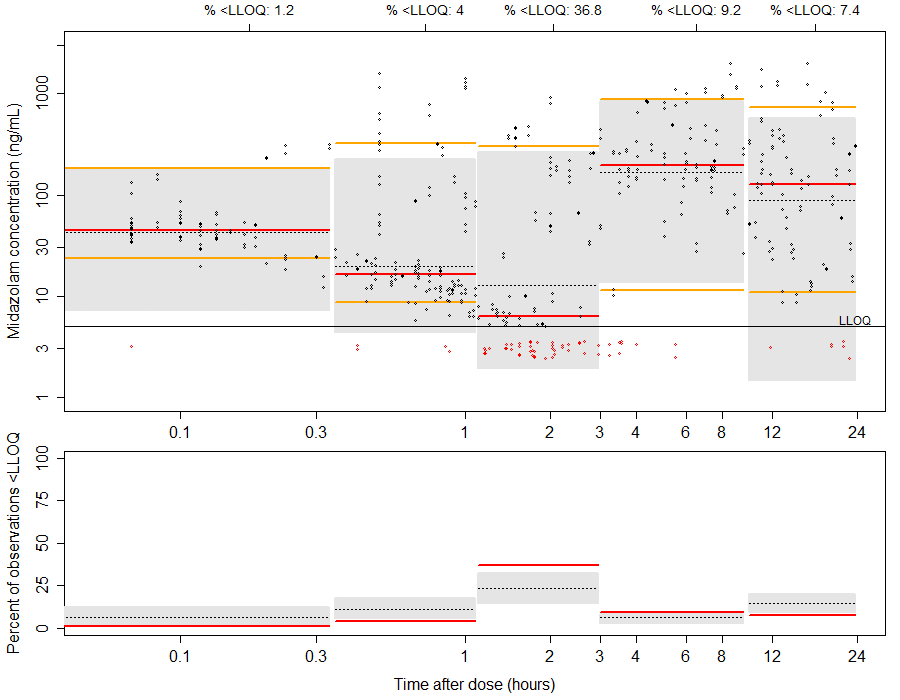


**Figure S-VIII:** Visual predictive check for midazolam observations from base PK model*.* Data have been divided into 5 bins of time after dose. Red lines show 50^th^ percentile of observed data in bin. Orange lines show 10^th^ and 90^th^ percentiles of observed data in bin. Dotted black lines show 50^th^ percentile of simulated data in bin. Grey shaded regions show range from 10^th^ to 90^th^ percentiles of simulated data in bin. Black circles show observed data. Red circles show the observations <LLOQ, which have been scattered around a concentration of 3 ng/mL (assay LLOQ is 5 ng/mL). Text at top of figure shows the percentage of observed data within each bin that are <LLOQ. The third bin has 36.8% of observations <LLOQ, hence the 10^th^ percentile of observed data is not calculable.


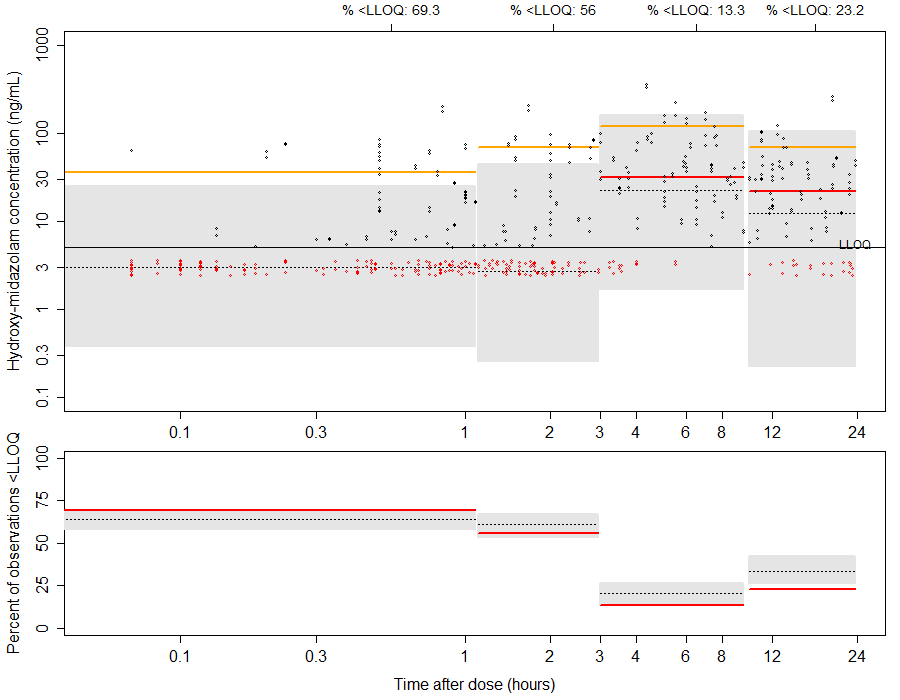


**Figure S-IX:** Visual predictive check for hydroxy-midazolam observations from base PK model*.* Data have been divided into 4 bins of time after dose. Red lines show 50^th^ percentile of observed data in bin. Orange lines show 10^th^ and 90^th^ percentiles of observed data in bin. Dotted black lines show 50^th^ percentile of simulated data in bin. Grey shaded regions show range from 10^th^ to 90^th^ percentiles of simulated data in bin. Black circles show observed data. Red circles show the observations <LLOQ, which have been scattered around a concentration of 3 ng/mL (assay LLOQ is 5 ng/mL). Text at top of figure shows the percentage of observed data within each bin that are <LLOQ. All bins have >10% of observations <LLOQ, hence the 10^th^ percentiles of observed data are not calculable. First 2 bins have >50% of observations <LLOQ, hence the 50^th^ percentiles of observed data are not calculable.

**
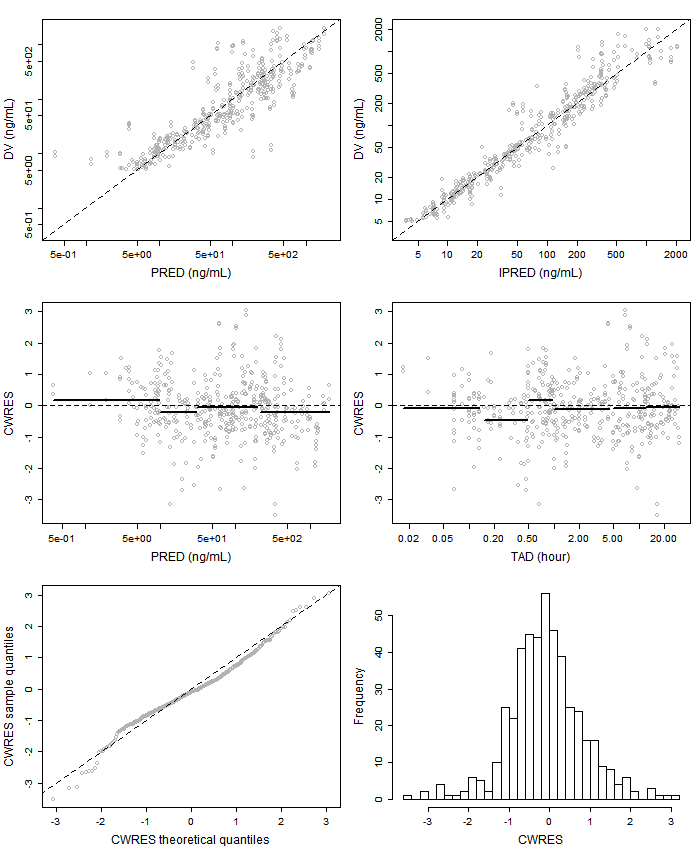
**

**Figure S-X:** Goodness of fit plots for midazolam observations from final PK model*.* All data included. Bold horizontal lines show the arithmetic mean of CWRES within the bin of independent variable (PRED, IPRED, TAD).


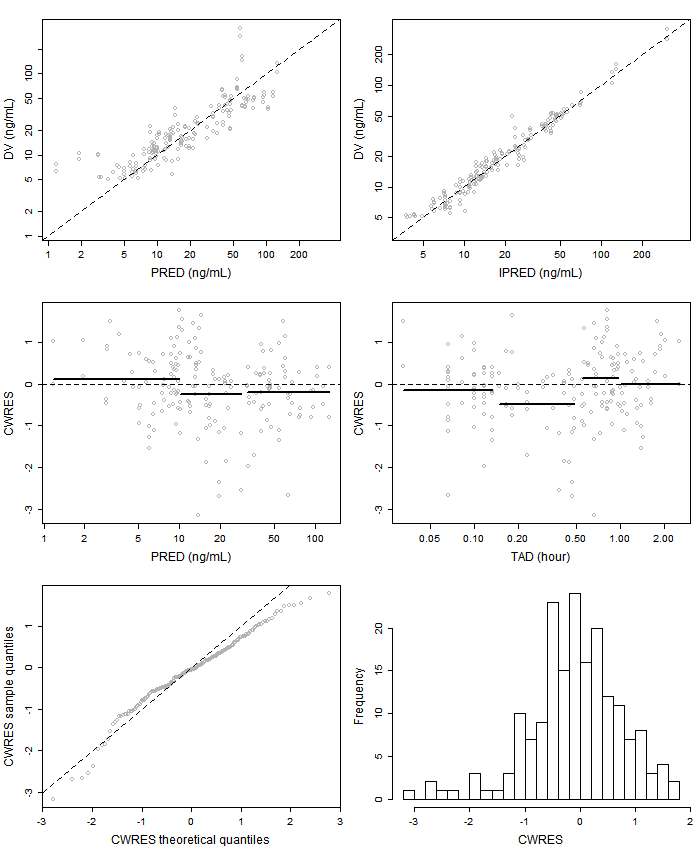


**Figure S-XI:** Goodness of fit plots for midazolam observations from final PK model*.* Only data from surgical cohort included. Bold horizontal lines show the arithmetic mean of CWRES within the bin of independent variable (PRED, IPRED, TAD).


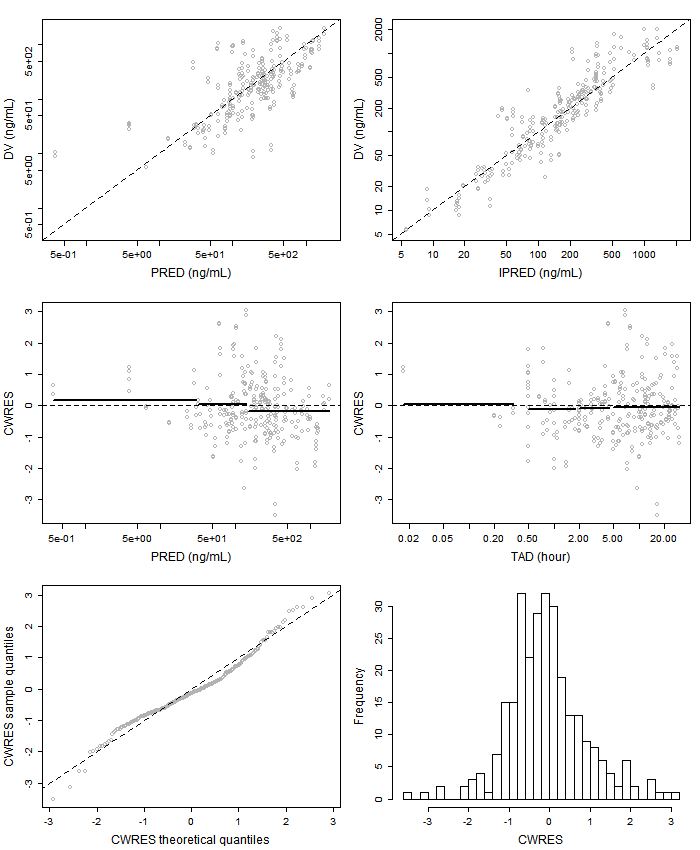


**Figure S-XII:** Goodness of fit plots for midazolam observations from final PK model*.* Only data from ICU cohort included. Bold horizontal lines show the arithmetic mean of CWRES within the bin of independent variable (PRED, IPRED, TAD).


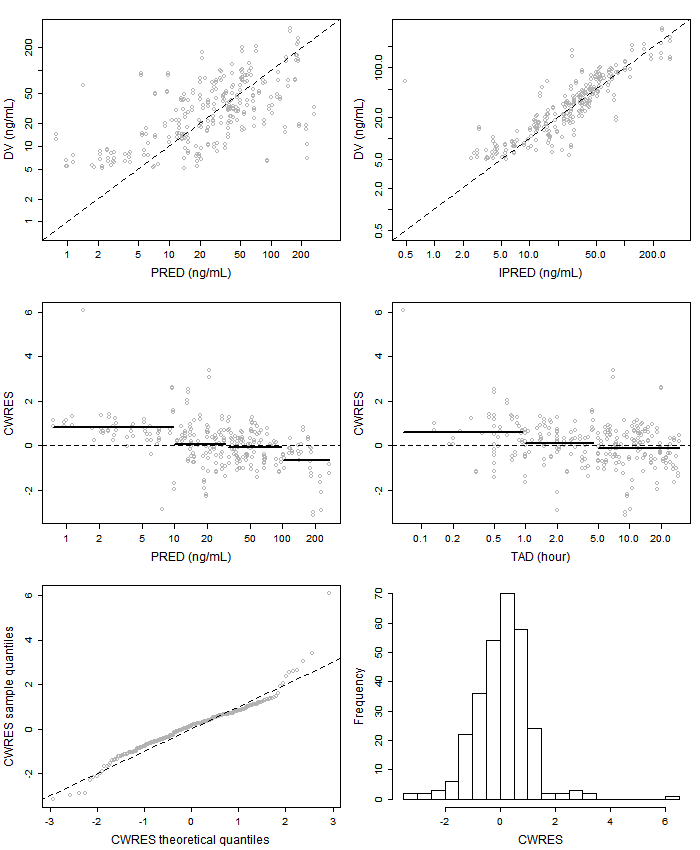


**Figure S-XIII:** Goodness of fit plots for hydroxy-midazolam observations from final PK model*.* All data included. Bold horizontal lines show the arithmetic mean of CWRES within the bin of independent variable (PRED, IPRED, TAD).


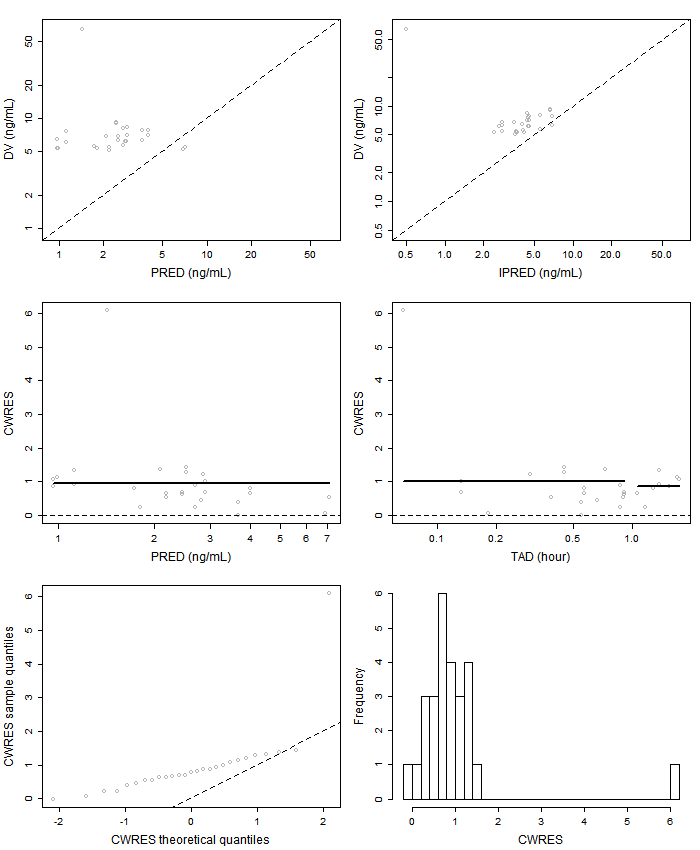


**Figure S-XIV:** Goodness of fit plots for hydroxy-midazolam observations from final PK model*.* Only data from surgical cohort included. Bold horizontal lines show the arithmetic mean of CWRES within the bin of independent variable (PRED, IPRED, TAD). Very few quantified hydroxy-midazolam observations in surgical cohort.


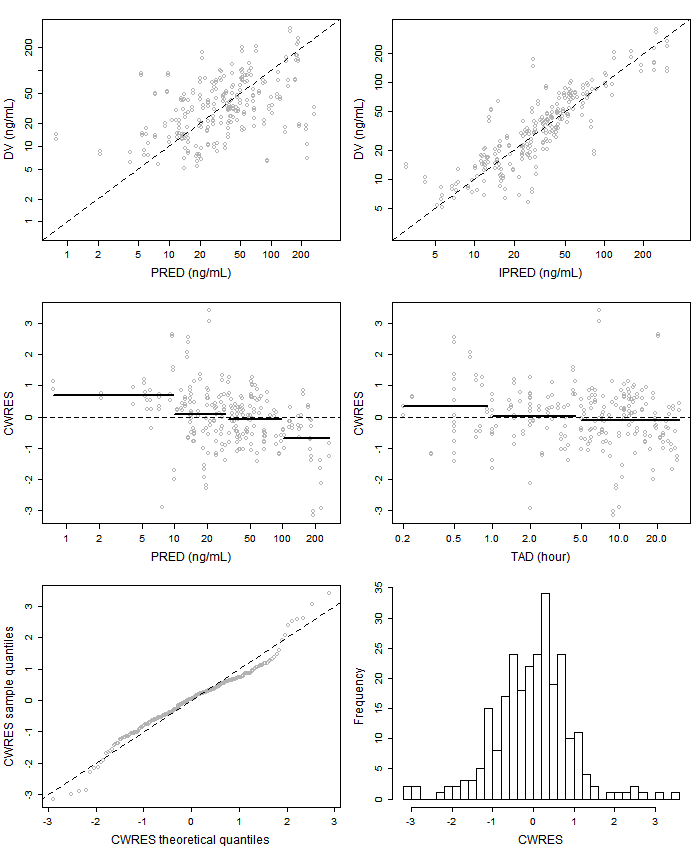


**Figure S-XV:** Goodness of fit plots for hydroxy-midazolam observations from final PK model*.* Only data from ICU cohort included. Bold horizontal lines show the arithmetic mean of CWRES within the bin of independent variable (PRED, IPRED, TAD).


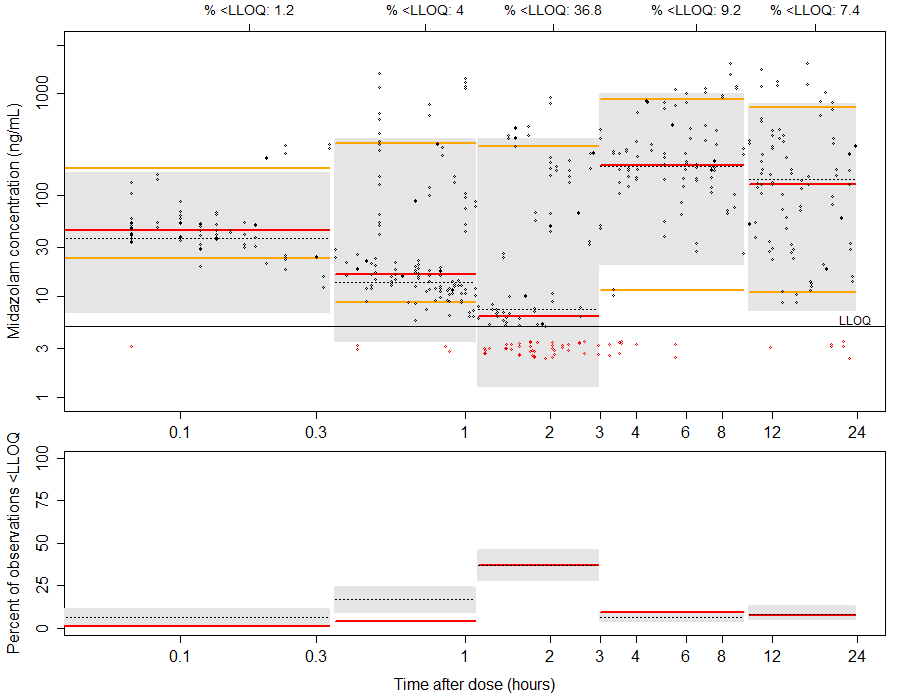


**Figure S-XVI:** Visual predictive check for midazolam observations from final PK model*.* Data have been divided into 5 bins of time after dose. Red lines show 50^th^ percentile of observed data in bin. Orange lines show 10^th^ and 90^th^ percentiles of observed data in bin. Dotted black lines show 50^th^ percentile of simulated data in bin. Grey shaded regions show range from 10^th^ to 90^th^ percentiles of simulated data in bin. Black circles show observed data. Red circles show the observations <LLOQ, which have been scattered around a concentration of 3 ng/mL (assay LLOQ is 5 ng/mL). Text at top of figure shows the percentage of observed data within each bin that are <LLOQ. The third bin has 36.8% of observations <LLOQ, hence the 10^th^ percentile of observed data is not calculable.


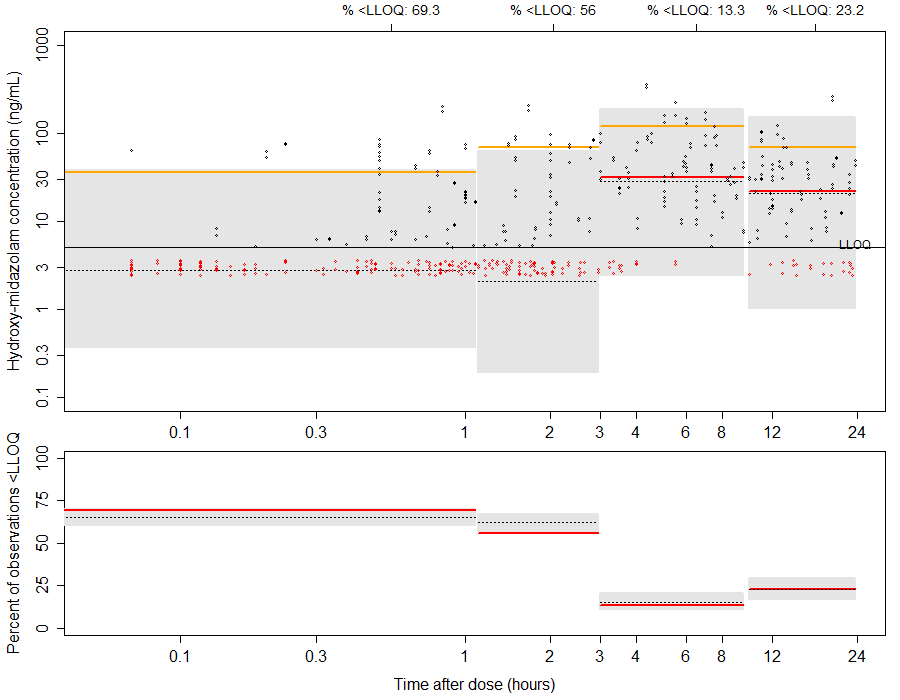


**Figure S-XVII:** Visual predictive check for hydroxy-midazolam observations from final PK model*.* Data have been divided into 4 bins of time after dose. Red lines show 50^th^ percentile of observed data in bin. Orange lines show 10^th^ and 90^th^ percentiles of observed data in bin. Dotted black lines show 50^th^ percentile of simulated data in bin. Grey shaded regions show range from 10^th^ to 90^th^ percentiles of simulated data in bin. Black circles show observed data. Red circles show the observations <LLOQ, which have been scattered around a concentration of 3 ng/mL (assay LLOQ is 5 ng/mL). Text at top of figure shows the percentage of observed data within each bin that are <LLOQ. All bins have >10% of observations <LLOQ, hence the 10^th^ percentiles of observed data are not calculable. First 2 bins have >50% of observations <LLOQ, hence the 50^th^ percentiles of observed data are not calculable.

**
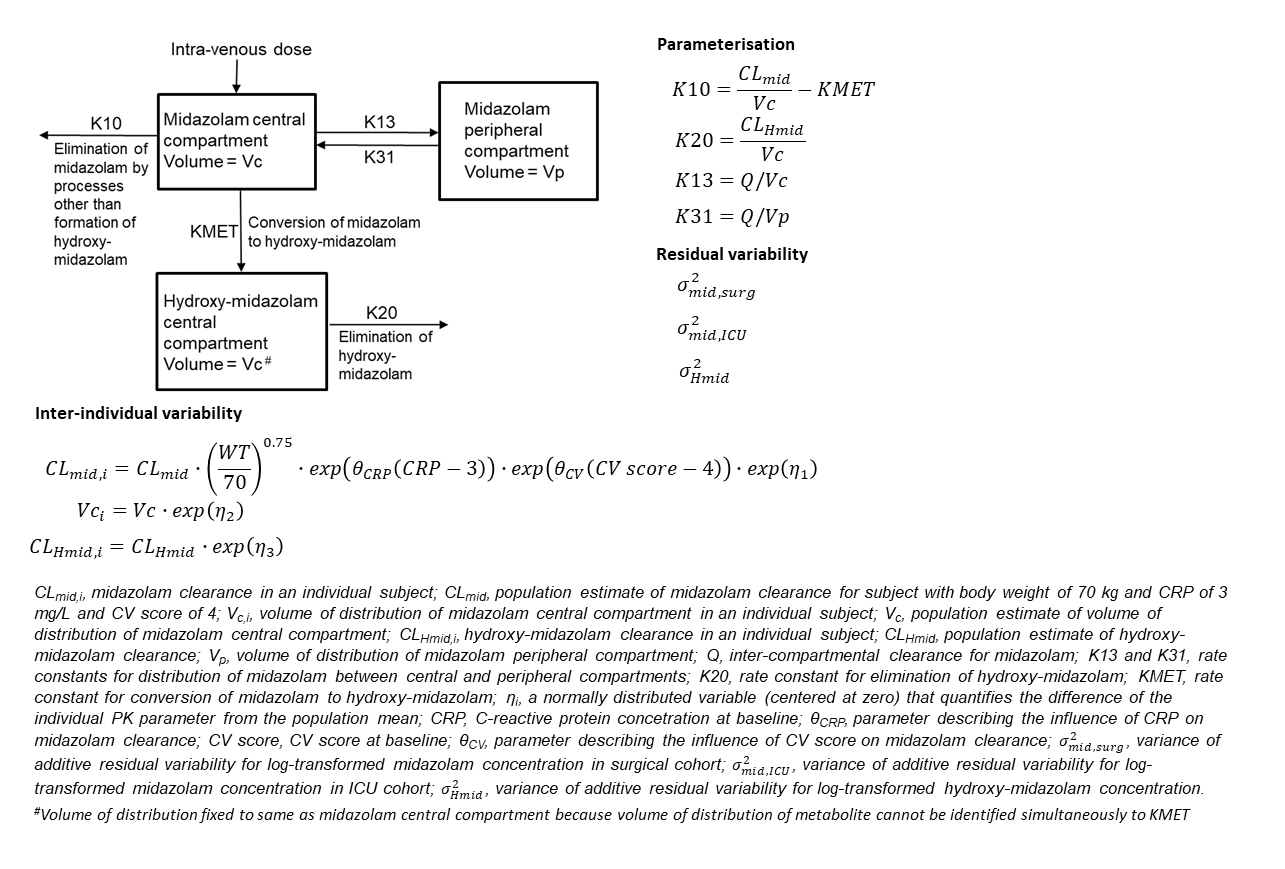
**

**Figure S-XVIII:** Compartmental and mathematical structure of final PK model.


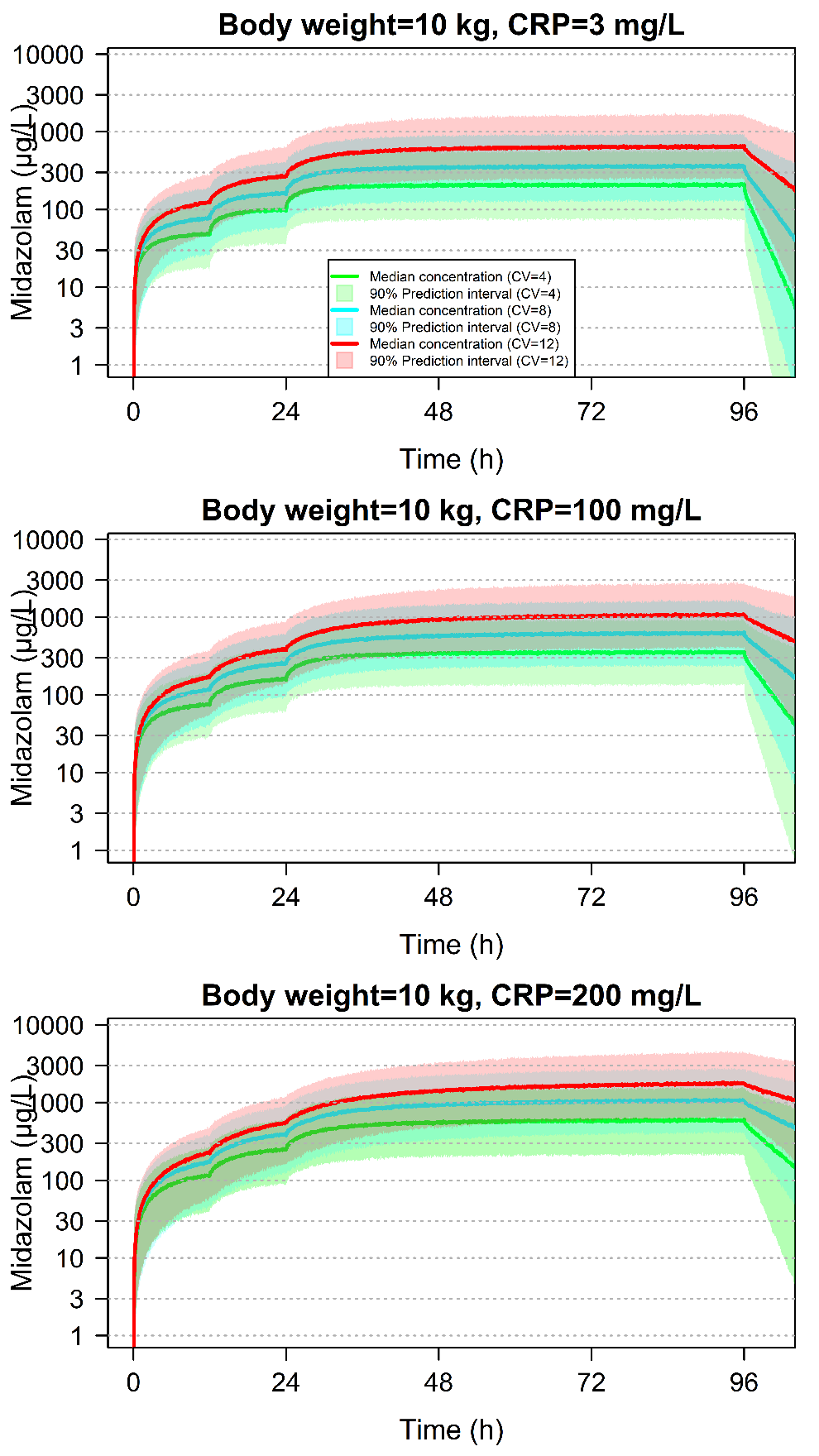


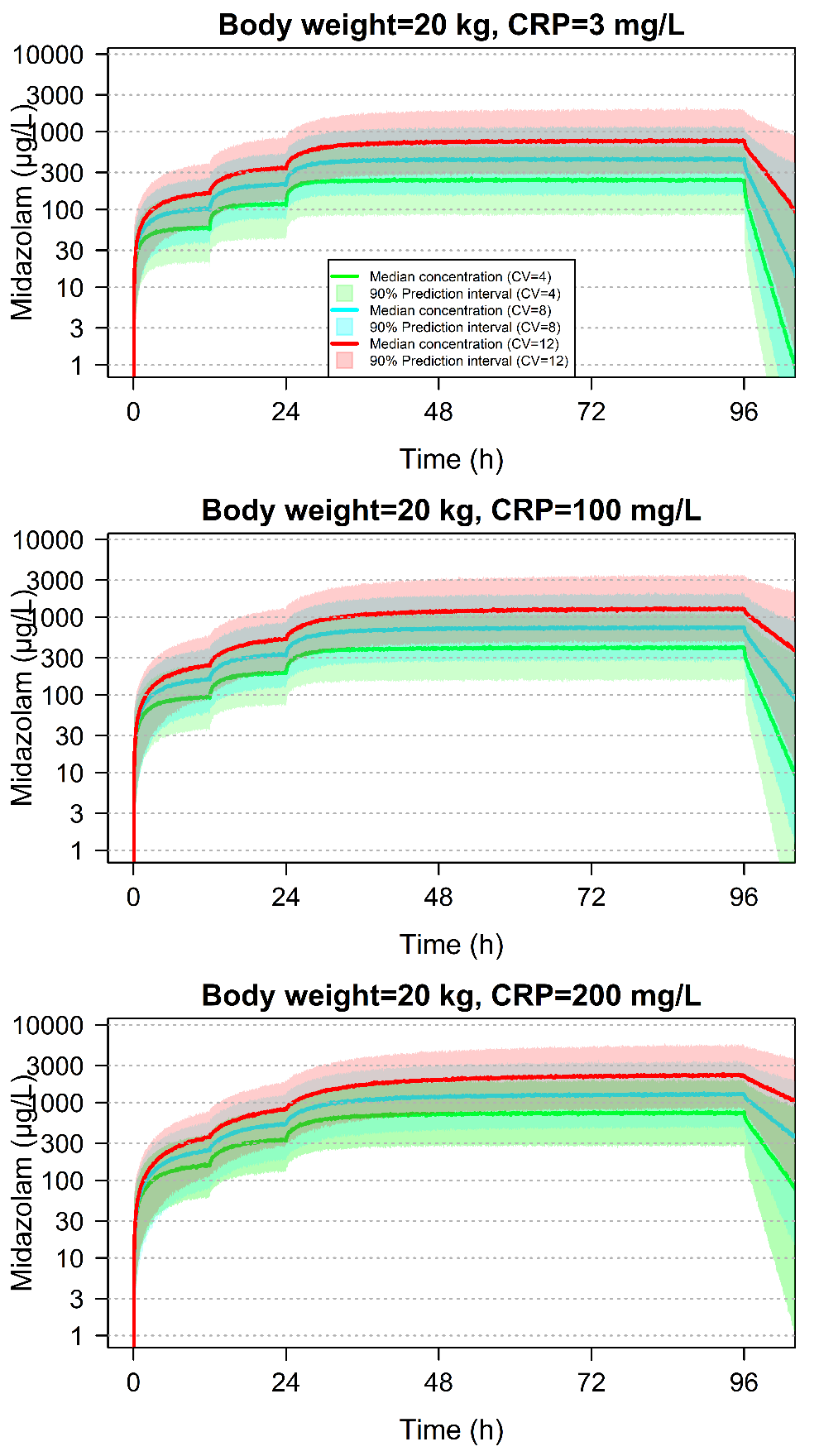


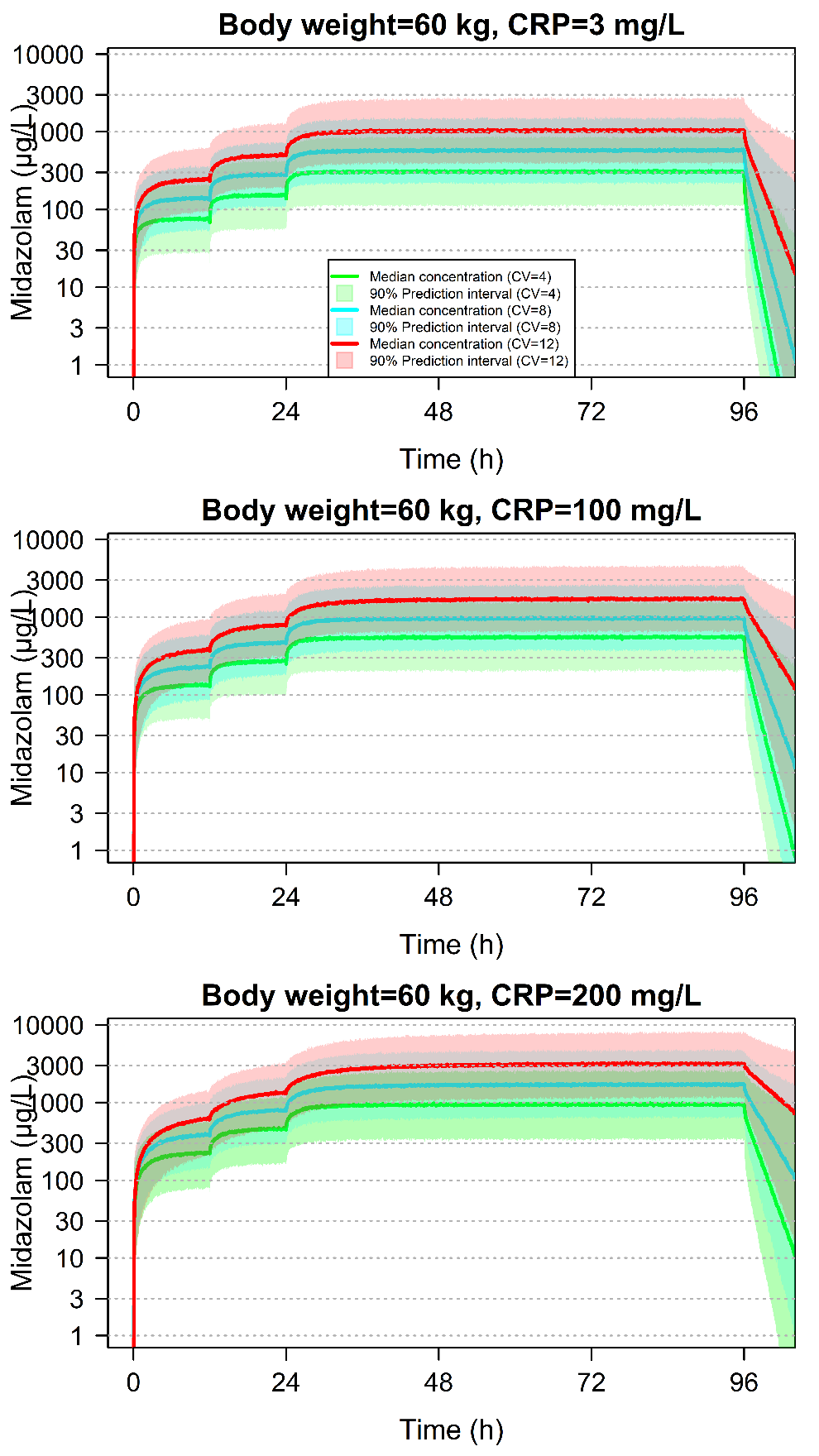


Figure S-XIX

Graphical displays of simulation outputs following a continuous midazolam infusion regimen: a bolus IV dose of 20 µg/kg immediately followed by continuous infusions of 60 µg/kg/h for 12 h, then 120 µg/kg/h for 12 h, then 240 µg/kg/h for 72 h. Body weights of the virtual patients were 10, 20 and 60 kg. CRP levels were 3, 100 and 200 mg/L, and CV scores were 4, 8 and 12. One panel of plots for each body weight, CRP varies within the 3 plots in each panel and CV score varies within each plot.

Table S1. Summary of simulations of steady state midazolam plasma concentrations using the final covariate model

|  |  |  | **Midazolam plasma concentration^a^ (µg/L)** | | |
| --- | --- | --- | --- | --- | --- |
| **Body weight (kg)** | **CRP (mg/L)** | **CV Score** | **LLCI^b^** | **Median** | **ULCI^c^** |
| 3.5 | 3 | 4 | 58.43 | 156.1 | 407.8 |
| 3.5 | 3 | 8 | 92.3 | 260.2 | 720.4 |
| 3.5 | 3 | 12 | 157.5 | 452.6 | 1168 |
| 3.5 | 100 | 4 | 95.5 | 253.7 | 655.6 |
| 3.5 | 100 | 8 | 172.7 | 461.2 | 1087 |
| 3.5 | 100 | 12 | 279.6 | 739.7 | 1761 |
| 3.5 | 200 | 4 | 159.4 | 450.9 | 1141 |
| 3.5 | 200 | 8 | 285.2 | 726.2 | 1786 |
| 3.5 | 200 | 12 | 450.6 | 1191 | 2652 |
| 10 | 3 | 4 | 74.0 | 212.8 | 530.4 |
| 10 | 3 | 8 | 131.2 | 363.2 | 932.0 |
| 10 | 3 | 12 | 245.1 | 659.9 | 1558 |
| 10 | 100 | 4 | 136.9 | 359.9 | 889.2 |
| 10 | 100 | 8 | 239.1 | 639.7 | 1574 |
| 10 | 100 | 12 | 413.0 | 1071 | 2731 |
| 10 | 200 | 4 | 205.7 | 601.4 | 1553 |
| 10 | 200 | 8 | 412.3 | 1044 | 2666 |
| 10 | 200 | 12 | 631.7 | 1757 | 4334 |
| 20 | 3 | 4 | 87.5 | 239.1 | 656.7 |
| 20 | 3 | 8 | 156.1 | 448.2 | 1139 |
| 20 | 3 | 12 | 275.0 | 764.9 | 1918 |
| 20 | 100 | 4 | 155.6 | 405.7 | 1105 |
| 20 | 100 | 8 | 277.0 | 738.8 | 2040 |
| 20 | 100 | 12 | 483.3 | 1283 | 3505 |
| 20 | 200 | 4 | 277.2 | 749.6 | 1923 |
| 20 | 200 | 8 | 490.9 | 1258 | 3278 |
| 20 | 200 | 12 | 841.7 | 2235 | 5408 |
| 60 | 3 | 4 | 112.5 | 310.8 | 820.6 |
| 60 | 3 | 8 | 213.8 | 571.9 | 1464 |
| 60 | 3 | 12 | 394.1 | 1033 | 2671 |
| 60 | 100 | 4 | 209.2 | 553.0 | 1561 |
| 60 | 100 | 8 | 375.3 | 955.7 | 2568 |
| 60 | 100 | 12 | 644.1 | 1685 | 4256 |
| 60 | 200 | 4 | 327.8 | 927.4 | 2592 |
| 60 | 200 | 8 | 651.2 | 1710 | 4563 |
| 60 | 200 | 12 | 1167 | 3166 | 8096 |
| ^a^ Simulated plasma concentrations at end of the final infusion dose (96 h)  ^b^ Lower limit of 90% prediction interval  ^c^ Upper limit of 90% prediction interval | | | | | |

Table S2. NONMEM model code for final PK model

; [DV] Plasma concentrations on log scale [log of conc units of ng/mL] (AMT has units of ng).

; [Data exclusions] See IGNORE commands

; [Structural model] 2 compartment model with linear elimination. Standard allometric scaling on CL without estimation of exponent. Different variance of residual error for icu / surgical.

; [Residual error] Additive.

; [IIV] IIV on CL, V1 and CLHY.

; [IIV covariance] ETA2 and ETA3.

; [Covariate model(s)] CRP influences CL. HD_FO influences CL.

; [Other] M3/IMP method used.

$PROBLEM 2 compartment PK model for midazolam and 1 compartment for hydroxy-midazolam, with IV dosing

$INPUT ID RECN CHT TIME TAD EVID XDV DV AMT RATE DUR CMT DVID DOSE SAMPLE MDVLLOQFLAG LLOQ HT HTX WT AGEY AGED GENDER ETHN DIAG CREAT ALT HCT_FO ALB_FO UREA_FO BILI_FO SODI_FO WCC_FO HD_FO INOT CRP VOL ENZ_IND ENZ_INH FLAG DROP

$DATA ../../Data/Midazolam_NM_v2.csv

IGNORE=@

IGNORE=(TIME.LT.0) ; Exclude records prior to first dose

IGNORE=(FLAG.EQ.2) ; Exclude subject who received 10 mg oral midazolam on day prior to first IV dose

IGNORE=(ID.EQ.13) ; Exclude subject with no quantified midazolam observations

IGNORE=(ID.EQ.43) ; Exclude subject with no quantified midazolam observations

IGNORE=(ID.EQ.52) ; Exclude subject with no quantified midazolam observations

IGNORE=(ID.EQ.58) ; Exclude subject with no quantified midazolam observations

IGNORE=(ID.EQ.65) ; Exclude subject with no quantified midazolam observations

IGNORE=(ID.EQ.83) ; Exclude subject with no quantified midazolam observations

$SUBROUTINE ADVAN5 TRANS1

$MODEL NCOMPARTMENTS=3

COMP=(CPT1 DEFOBS DEFDOSE) ; central compartment for midazolam

COMP=(CPT2) ; central compartment for hydroxymidazolam

COMP=(CPT3) ; Peripheral compartment for midazolam

$PK

MXSTEP=1000000

; CRP influences CL

COV1=CRP

COVTH1=THETA(9)

COVSC1=3

COVINF1=exp(COVTH1*(COV1-COVSC1))

; HD_FO influences CL

COV2=HD_FO

COVTH2=THETA(10)

COVSC2=4

COVINF2=exp(COVTH2*(COV2-COVSC2))

MU_1 = LOG(1000*THETA(1))

CL = EXP(MU_1 + ETA(1))*COVINF1*COVINF2*(WT/70)**0.75 ; Clearance of midazolam [units: L/h]

MU_2 = LOG(1000*THETA(2))

V1 = EXP(MU_2 + ETA(2)) ; Volume of central compartment for midazolam [units: L]. This volume is also applied to hydroxymidazolam

D1 = THETA(3) ; Duration of the bolus "infusion" dose [units: h]

Q = 1000*THETA(4) ; Inter-compartmental clearance of midazolam [units: L/h]

V2 = 1000*THETA(5) ; Volume of peripheral compartment of midazolam [units: L]

MU_3 = LOG(1000*THETA(6))

CLHY = EXP(MU_3 + ETA(3)) ; Clearance of hydroxymidazolam [units: L/h]

KMET = THETA(7) ; Apparent rate constant for metabolite formation

MSC = THETA(8) ; Scaling factor for concentrations from mitra assay

N1 = 0

IF(DVID.EQ.2) N1 = 1

K10 = CL/V1 - KMET

K12 = KMET

K20 = CLHY/V1

K13 = Q/V1

K31 = Q/V2

N2=0

IF(CHT.EQ.1) N2=1

$ERROR

IRES=0

IWRES=0

C1 = (1 + N1*MSC)*(A(1)/V1)

IF(C1.LE.0) C1=1

C2 = (1 + N1*MSC)*(A(2)/V1)

IF(C2.LE.0) C2=1

IPRED = 0

W1 = N2*SQRT(SIGMA(1,1)) + (1-N2)*SQRT(SIGMA(2,2)) ; Weights for midazolam concentrations, which depend on icu or surgical cohort

W2 = SQRT(SIGMA(3,3)) ; Weights for hydroxy-midazolam observations

IF(LLOQFLAG.EQ.0.AND.EVID.EQ.0.AND.CMT.EQ.1) THEN

; quantified midazolam observation

F_FLAG=0

IPRED = LOG(C1)

Y = IPRED + N2*EPS(1) + (1-N2)*EPS(2)

IRES = DV - IPRED

IWRES = IRES/W1

ENDIF

IF(LLOQFLAG.EQ.0.AND.EVID.EQ.0.AND.CMT.EQ.2) THEN

; quantified hydroxymidazolam observation

F_FLAG=0

IPRED = LOG(C2)

Y = IPRED + EPS(3)

IRES = DV - IPRED

IWRES = IRES/W2

ENDIF

IF (LLOQFLAG.EQ.1.AND.EVID.EQ.0.AND.CMT.EQ.1) THEN

; BLQ midazolam observation

F_FLAG=1

LN_LLOQ = LOG(LLOQ)

IPRED = LOG(C1)

Y=PHI((LN_LLOQ-IPRED)/W1) ; Likelihood calculation for observation below LLOQ

MDVRES=1

ENDIF

IF (LLOQFLAG.EQ.1.AND.EVID.EQ.0.AND.CMT.EQ.2) THEN

; BLQ hydroxymidazolam observation

F_FLAG=1

LN_LLOQ = LOG(LLOQ)

IPRED = LOG(C2)

Y=PHI((LN_LLOQ-IPRED)/W2) ; Likelihood calculation for observation below LLOQ

MDVRES=1

ENDIF

$THETA

(0,20,10000) ;[TH1] CL [L/h]

(0,7,10000) ;[TH2] V1 [L]

0.0333 FIX ;[TH3] D1 [h]

(0,30,10000) ;[TH4] Q [L/h]

(0,20,10000) ;[TH5] V2 [L]

(0,5,10000) ;[TH6] CLHY [L/h]

(0,1,10000) ;[TH7] KMET [h-1]

0 FIX ;[TH8] MSC [NA]

(-1,0.01,1) ;[TH9] COVTH1

(-1,0.01,1) ;[TH10] COVTH2

$OMEGA

0.3 ; Variance of IIV on CL

$OMEGA BLOCK(2)

0.5 ; Variance of IIV on V1

0.05 0.3 ; Variance of IIV on CLHY

$SIGMA

0.1 ; Variance of residual variability for midazolam observations in surgical data

0.1 ; Variance of residual variability for midazolam observations in icu data

0.1 ; Variance of residual variability for hydroxy-midazolam observations surgical and icu data

$EST METHOD=IMP INTERACTION LAPLACIAN EONLY=0 ISAMPLE=300 NITER=1000 CTYPE=3 NOABORT

GRD=SG(1,2) NOTHETABOUNDTEST PRINT=1

$EST METHOD=IMP INTERACTION LAPLACIAN EONLY=1 ISAMPLE=3000 NITER=10 SEED=9432 NOABORT

GRD=SG(1,2) PRINT=1

$COV PRINT=E MATRIX=S SIGL=12

$TABLE ID CHT RECN TIME TAD MDV EVID DVID CMT AMT RATE DUR DOSE AGEY WT

XDV C1 C2 DV IPRED PRED

LLOQFLAG CWRES IRES IWRES

HT HTX WT AGEY AGED GENDER ETHN DIAG CREAT ALT HCT_FO ALB_FO UREA_FO

BILI_FO SODI_FO WCC_FO HD_FO INOT CRP VOL ENZ_IND ENZ_INH

ETA1 ETA2 ETA3

CL V1 FLAG NOAPPEND NOPRINT ONEHEADER FILE=run037.tab
